# Selective inhibitors targeting Fis1/Mid51 protein-protein interactions protect against hypoxia-induced damage in cardiomyocytes

**DOI:** 10.3389/fphar.2023.1275370

**Published:** 2023-12-21

**Authors:** Mulate Zerihun, Nir Qvit

**Affiliations:** The Azrieli Faculty of Medicine in the Galilee, Bar-Ilan University, Safed, Israel

**Keywords:** mitochondrial fission 1 (Fis1), mitochondrial dynamics 51 kDa (Mid51), peptide, inhibitor, protein-protein interaction (PPI), cardiovascular diseases (CVDs)

## Abstract

Cardiovascular diseases (CVDs) are the most common non-communicable diseases globally. An estimated 17.9 million people died from CVDs in 2019, representing 32% of all global deaths. Mitochondria play critical roles in cellular metabolic homeostasis, cell survival, and cell death, as well as producing most of the cell’s energy. Protein–protein interactions (PPIs) have a significant role in physiological and pathological processes, and aberrant PPIs are associated with various diseases, therefore they are potential drug targets for a broad range of therapeutic areas. Due to their ability to mimic natural interaction motifs and cover relatively larger interaction region, peptides are very promising as PPI inhibitors. To expedite drug discovery, computational approaches are widely used for screening potential lead compounds. Here, we developed peptides that inhibit mitochondrial fission 1 (Fis1)/mitochondrial dynamics 51 kDa (Mid51) PPI to reduce the cellular damage that can lead to various human pathologies, such as CVDs. Based on a rational design approach we developed peptide inhibitors of the Fis1/Mid51 PPI. *In silico* and *in vitro* studies were done to evaluate the biological activity and molecular interactions of the peptides*.* Two peptides, CVP-241 and CVP-242 were identified based on low binding energy and molecular dynamics simulations. These peptides inhibit Fis1/Mid51 PPI (-1324.9 kcal mol^−1^) in docking calculations (CVP-241, -741.3 kcal mol^−1^, and CVP-242, -747.4 kcal mol^−1^), as well as in *vitro* experimental studies Fis1/Mid51 PPI (K_D_ 0.054 µM) Fis1/Mid51 PPI + CVP-241 (K_D_ 3.43 µM), and Fis1/Mid51 PPI + CVP-242 (K_D_ 44.58 µM). Finally, these peptides have no toxicity to H9c2 cells, and they increase cell viability in cardiomyocytes (H9c2 cells). Consequently, the identified inhibitor peptides could serve as potent molecules in basic research and as leads for therapeutic development.

## 1 Introduction

Cardiovascular disease (CVD) is a group of diseases that affect the heart and blood vessels. CVDs account for the majority of deaths in the United States (US) and around the world. 18 million CVD deaths occurred worldwide in 2019, accounting for 32% of all deaths. Based on estimates, CVDs will cause 25 million deaths worldwide by 2030. According to the American Heart Association (AHA), the total direct (medical) and indirect (lost productivity) costs of CVDs in the US alone were $555 billion in 2016 and are expected to exceed $1 trillion by 2035. Mitochondria are membrane-bound organelles that generate most of the chemical energy needed to power cell biochemical reactions, which is stored in a small molecule called adenosine triphosphate (ATP). They also contribute to the maintenance of cellular metabolic homeostasis, as well as cell survival and death (Cogliati et al., 2016; Formosa and Ryan, 2018). The mitochondrial organelle is also an important therapeutic target for a variety of human diseases (Veloso et al., 2019), and mitochondrial dynamics imbalances have been associated with a variety of human diseases, including neurodegenerative, metabolic, and CVDs (Akhmedov et al., 2015; Suárez-Rivero et al., 2016). The dynamics of mitochondria are governed by two processes: fusion, which joins two mitochondria together, and fission, which allows a mitochondrion to divide into two mitochondria (Detmer and Chan, 2007). To maintain mitochondrial network homeostasis, a balance in mitochondrial dynamics is necessary (Eisner et al., 2018; Simula et al., 2019).

Mitochondrial fission protein 1 (Fis1) was first discovered in *Saccharomyces cerevisiae* via complementation screens, where it is the sole recruitment factor on the outer mitochondrial membrane (OMM) (Mozdy et al., 2000; Youle and Narendra, 2011). Human orthologues of this protein, known as hFis1, are members of mitochondrial complexes that promote fission of mitochondria (James et al., 2003; Friedman and Nunnari, 2014). This protein is attached to the OMM by its C-terminal tail, exposing an N-terminal domain comprised of two tetratricopeptide repeats (TPRs) to the cytoplasm, which are common PPI domains (Dohm et al., 2004; Suzuki et al., 2005; Zhang and Chan, 2007; Ihenacho et al., 2021; Egner et al., 2022).

Dynamin-related protein 1 (Drp1), a large guanosine triphosphate (GTP)ase, is the key mediator of mitochondrial fission. During mitochondrial division Drp1 interacts with the OMM-localized adaptor proteins such as mitochondrial fission factor (Mff), mitochondrial dynamics protein of 49 and 51 kDa (MiD49/51), as well as Fis1 (Losón et al., 2013; Xian and Liou, 2021). Fis1 was the first adaptor identified for mitochondrial fission, initially in yeast (Cerveny and Jensen, 2003) and subsequently determined to mediate human mitochondrial and peroxisomal fission (Schrader et al., 2012), and it is the only recruiter conserved across mitochondrial-containing species (Zhao et al., 2013).

After initially being identified as the Smith-Magenis syndrome chromosome region candidate gene 7 (SMCR7) and SMCR7-like (SMCR7L) proteins, Mid51 and Mid49 have been renamed mitochondrial dynamics proteins of 49 kDa (aka mitochondrial elongation factor 2 (Mief2)) and 51 kDa (aka mitochondrial elongation factor 1 (Mief1)) due to their mitochondrial activity and size (Palmer et al., 2011; Samangouei et al., 2018). Despite general agreement that Mid49 and Mid51 play a crucial role in mitochondrial dynamics, the question remains whether they belong to the mitochondrial fusion or fission protein family. While Mid51 recruits Drp1 to the mitochondrial surface to execute fission (Palmer et al., 2013), it was demonstrated that Mid51 suppresses Drp1 function and promotes mitochondrial elongation by suppressing mitochondrial fission (Zhao et al., 2011; Palmer et al., 2013; Atkins et al., 2016).

Interestingly, Zhao *et al.* demonstrated that Fis1 was co-immunoprecipitated with Mid51 and proposed that Fis1/Mid51 PPI is independent of Mid51 interaction with Drp1 and its mitochondrial localization (Zhao et al., 2011). And a study by Wong *et al.* suggested that Fis1/Mid51 PPI is important for Drp1 GTP hydrolysis machinery at mitochondria-lysosome contact sites (Wong et al., 2022). Finally, CVD appears to be impacted by Fis1 and Mid51 proteins, and there is some evidence that Fis1 and Mid51 PPIs are promising targets for the regulation and treatment of pathological fission processes and CVD (Vásquez-Trincado and Schinkel, 2016).

Protein-protein interactions (PPIs) are an integral component of all life events and cellular activities, regulating cell life and death, and mediating a variety of biochemical reactions (Li et al., 2020; Luck et al., 2020). Consequently, PPIs have become the “holy grail” of contemporary basic research and have emerged as promising therapeutic targets for a wide range of medical conditions (Davenport et al., 2020). The rational design of PPI inhibitors is considered to be a promising area for basic research and drug discovery in modern pharmacology (Mabonga and Kappo, 2019). Due to their physicochemical characteristics, PPIs pose a challenge for conventional small-molecule drugs, including their large, featureless contact patches without hydrophobic pockets for binding (Wang et al., 2021). A more effective method of targeting PPIs may be the use of therapeutic proteins (aka monoclonal antibodies), but such agents have a high production cost and are not cell permeable. The peptides are placed between small molecules and large antibodies, and they are large enough to effectively inhibit PPIs. As an alternative to antibodies, they exhibit faster clearance and higher specificity than small molecules (Kornfeld et al., 2018; Anand et al., 2023). Aside from this, they can also be chemically synthesized, which results in a reduction in costs and an improvement in batch-to-batch reproducibility (d'Orlyé et al., 2021). In light of the above considerations, peptides that inhibit PPIs have a wide range of potential applications as tools for studying biological activity and potential therapeutic targets. It has been shown in previous studies that peptides designed to inhibit Fis1/Drp1 and Mff/Drp1 PPIs may provide therapeutic benefits in animal models of neurodegenerative diseases (Qi et al., 2013; Joshi et al., 2019) and CVDs (Disatnik et al., 2013).

In the preclinical stage of drug discovery, computer-based drug design provides a cost-effective and efficient method of identifying potential drug candidates (Salman et al., 2021; Liu et al., 2022). Computer-aided drug design utilizes virtual screening as one of its major methods (Khan et al., 2021). As a result of the availability of three-dimensional (3D) crystal structures of protein receptors, molecular docking simulation techniques are widely used in virtual screening (Gentile et al., 2022). Drug discovery has been enhanced by computer-aided drug design and integration with experimental routines (Guardiola et al., 2021). The majority of peptide structure-based design initiatives incorporate structural motifs such as helix binding domains to mimic a specific epitope found in one of the PPI partners (Zheng et al., 2015; Perez et al., 2021). The use of molecular docking techniques is critical in predicting binding affinity and validating protein-peptide complexes (Menchon et al., 2018).

It was suggested that Fis1/Mid51 PPI results in increased mitochondrial pathological fission (Zhao et al., 2011; Vásquez-Trincado and Schinkel, 2016; Liu et al., 2023). Moreover, imbalanced mitochondrial dynamics were demonstrated to lead to a number of human pathologies, such as CVDs (Zerihun et al., 2023). Therefore, an inhibitor of this interaction may decrease mitochondrial fission and potentially have therapeutic utility. Our studies investigated this hypothesis by using a rational design approach, *in silico* simulations, and molecular docking approach to identify novel peptide inhibitors of Fis1/Mid51 PPI. We developed CVP-241 and CVP-242 that mediate cardioprotection in a cardiomyocyte model of ischemia-reperfusion injury. These peptides are potential therapeutic agents for improving mitochondrial homeostasis and cell fate.

## 2 Materials and methods

### 2.1 Sequence alignments and rational designs of peptides

Sequences from different species were aligned using the LALIGN server (accessed on January 2023), using Fis1 proteins (*Homo sapiens* (Q9Y3D6), *Mus musculus* (Q9CQ92), *Rattus norvegicus* (P84817), and *Drosophila melanogaster* (B7YZT2); as well as Mid51 proteins (*Homo sapiens* (Q9NQG6), *Mus musculus* (Q8BGV8), *Rattus norvegicus* (Q5XIS8), and *Xenopus tropicalls* (Q52MA5)).

### 2.2 Prediction of physicochemical properties, pharmacokinetics, drug-likeness, toxicity, and biological activity

The ProtParam web server calculates physical and chemical parameters, such as the theoretical isoelectric point (pI), length, number of positive and negative residues, and hydrophobicity of inhibitor peptides (Tetko et al., 2005). In addition, a peptide property calculator (https://pepca lc.com) and SwissADME (http://www.swissadme.ch) were used to determine the molecular mass and topological polar surface area (TPSA). Using the “SMILES” feature of the NovoPro server (https://www.novoprolabs.com), the amino acid sequences of the designed peptides were converted into SMILES (Daina et al., 2017). Several drug-like properties, including the molecular weight of the compound (MW), the consensus octanol-water partition coefficient (C Log Po/w), the number of hydrogen bond donors (nON), and the number of hydrogen bond acceptors (nOHNH), were calculated by the SwissADME tool, Swiss Institute of Bioinformatics (https://www.swissadme.ch, accessed on January 2023) and investigated for violations of Lipinski’s rule of five and pharmacokinetics properties (Lipinski, 2004; Daina et al., 2017). In addition to blood-brain barrier penetration (Cbrain/Cblood), ADMETLAB was used to calculate pharmacokinetic indicators, including intestinal absorption (%), skin permeability (logKp, cm/hour), water solubility (mg/L), plasma protein binding (%), mutagenic and carcinogenic effects, as well as potential inhibition of CYP3A4 (cytochrome P450 3A4) (Didziapetris et al., 2003; Wang et al., 2016). ToxinPred (http://crdd.osdd.net/raghava/toxinpred) was used to predict the potential toxicity of the peptides (Gupta et al., 2013). A cheminformatics online server, Molinspiration (https://www.molinspiration.com), was used to predict the biological activity of the peptides.

### 2.3 Molecular docking and binding prediction analysis

An online docking system called ClusPro and Pymol structures were used to visualize the structure of the peptides. A PDB/Alphafold file and the structural information of the target proteins were obtained from the Protein Data Bank (http://www.rcsb.org/pdb). Following the building of the peptide through the pymol-assisted (https://pymolwiki.org/index.php/Fab) and ClusPro 2.0 (Docking for proteins and peptides), the predicted secondary structure was calculated using chou-Fasman method (http://www.biogem.org/tool/chou-fasman/). Affinity energy values were used to evaluate all docking modes generated. Based on the affinity energy, ClusPro calculated the estimated dissociation constant (Ki). Using the software, hydrogen bonds, hydrophobic interactions, and electrostatic interactions between protein and peptide residues were observed (Pande et al., 2021). Moreover, the protein data bank (PDB) format was saved in the protein data bank (BIOVIA Discovery Studio Visualizer) format and applied to HPEPDOCK analysis (Gupta et al., 2013). BIOVIA Discovery Studio Visualizer was applied to visualize protein-peptide docking models. LigPlot + v.2.2 was used to visualize the two-dimensional (2D) diagram of docked models with relatively lower (more negative) docking scores (Laskowski and Swindells, 2011). We examined molecular interactions between peptides and proteins, including hydrogen bonds, hydrophobic interactions, salt bridges, and external bonds.

### 2.4 Peptides synthesis, purification, and characterization

The peptides were chemically synthesized using the fluorenyl methoxycarbonyl (Fmoc)/tert-butyl (tBu) method (Ben-Uliel et al., 2022), using a fully automated parallel peptide synthesizer (Syro I, Biotage, Uppsala, Sweden). The solvents and reagents were all commercially available and were not purified in any way. Piperidine, diethyl ether, N, N Diisopropylethylamine (DIEA), Trifluoroacetic acid (TFA), and Water (HPLC grade) were obtained from Bio-Lab (Jerusalem, Israel); Acetonitrile (ACN) (HPLC grade) was acquired from J.T. Baker (Poland); Acetic anhydride was obtained from Daejung (Gimhae-si, Korea); Triisopropylsilane (TIS) was purchased from Acros organics (Branchburg, NJ, United States); dimethylformamide (DMF) was purchased from Carlo Erba (Val De Reuil, France); Oxyma Pure was contributed by Luxembourg Bio Technologies Ltd. (Ness Ziona, Israel); N,N-Diisopropylcarbodiimide (DIC) was purchased from Angene International Limited (Nanjing, China); Fmoc Rink amide MBHA resin was purchased from AnaSpec (substitution 0.67 mmol/g, Fremont, CA, United States); Fmoc-protected amino acids were purchased from Ontores Biotechnologies (Hangzhou, China). Side chains of the amino acids used in the synthesis were protected as follows: tert-Butyloxycarbonyl (BOC) (Lys/Met), tert-butyl (tBu) (Ser/Thr/Tyr/Glu), t-butyl ester (OtBu) (Asp), 2,2,4,6,7-Pentamethyldihydrobenzofuran-5-sulfonyl (Pbf) (Arg), 4-methyltrityl (Mtt) (Lys), and triphenylmethy (Trt) (Asn/Gln/His).

Fmoc deprotection was carried out in two steps at 75 °C using piperidine (40%) in DMF solution for three and 12 minutes, respectively. Coupling reactions were carried out by repetition of the following cycle conditions: 45 min at 75 °C with DIC (0.2 M) in DMF, Oxyma Pure (0.2 M) in DMF, and amino acid (0.2 M) in DMF. The coupling and Fmoc deprotection steps were monitored whenever necessary using small cleavages. In order to achieve anhydride coupling, the following cycle conditions were repeated: 30 min at room temperature, using anhydride (10 eq)/DIEA (10 eq)/peptide (1 eq) in DMF. In the final step of the synthesis, the peptide was cleaved from the resin, and its amino acid side chains were deprotected with a pre-cooled mixture of TFA/TIS/H_2_O solution (90:2.5:2.5 v/v/v) for 3 hours at room temperature. A stream of compressed air was used to evaporate the solvents and remove the resin. After precipitating the crude products with diethyl ether, centrifugation was carried out to collect the residue, which was then dissolved in CH_3_CN/H_2_O (30:70) and lyophilized.

Reverse-phase high-pressure liquid chromatography (1260 Infinity II LC System) was used to analyze the products. The system was equipped with a G7129A 1260 vial sampler, a G7111B 1260 quaternary pump, a G7115A 1260 DAD detector (diode array detector) WR, a G1364C 1260 FC-AS and a G1330B 1290 thermostat from Agilent (Santa Clara, California, United States). A Luna 5 µm C18(2) 100-mm (Phenomenex, Torrance, CA, United States) column (Phenomenex, Torrance, California, United States) was used. A solvent system containing 0.1% TFA in H_2_O was used, and a solvent system containing 0.1% TFA in CH_3_CN was used. We applied a linear gradient of 5%–95% B over 45 min and detected the samples at 214 nm and 254 nm. The synthesis products were purified on a Luna 5 µm C18(2) 100 Å (250 × 10 mm) column (Phenomenex, Torrance, CA, United States) at 4.7 ml/min. There were two solvent systems used, A (H_2_O with 0.1% TFA) and B (CH_3_CN with 0.1% TFA). The separation was performed using a linear gradient of 5%–95% B in 45 min, and the detection wavelengths were 214 nm and 254 nm. We analyzed the peptides using matrix-assisted laser desorption/ionization mass spectrometry (MALDI-MS) (autoflex^®^ maX, Bruker, Billerica, MA, United States). Based on the results of the *in vitro* binding assay, the selected peptides were synthesized as single polypeptides containing TAT carriers from N-terminus to TAT-spacer (Gly-Gly) to cargo to C-terminus.

### 2.5 Protein expression and purification

The two recombinant proteins, Fis1 and Mid51 were expressed using the *Escherichia coli* (*E. coli*) Rosetta bacterial expression system. All constructs were transformed into *E. coli* XL1-Gold strain Stratagene (San Francisco, CA, United States) using heat shock method, followed by cultivation and plasmid DNA purification using the QIAprep minispin kit (QIAGEN, Hilden, Germany). Transformed bacteria was cultured at 37 °C until OD_600_ = 0.6 nm and induced with 0.1 mM Isopropyl *β*-d-1-thiogalactopyranoside (IPTG) for 16 h at 16°C–18°C (overnight). After cultivated, the cells were collected by centrifugation, washed with cold phosphate-buffered saline (PBS) and stored at -20 °C. The collected pellets were suspended in lysis buffer and using high-pressure homogenizer (Avestin) followed by centrifugation at 40,000 g for 1 h.

Finally, the supernatant was applied to resin packed Ni-NTA column equilibrated with wash buffer. After washing with washing buffer, the proteins were eluted with elution buffer and the eluted protein was purified using affinity chromatography. After the chromatography, the eluted protein fractions were separated using SDS-PAGE followed by western blot. The proteins were detected using specific primary antibody anti-Mid 51 (SC-514135, Santa Cruz Biotechnology, TX, United States) and anti-Fis1 (SC-376447, Santa Cruz Biotechnology, TX, United States). Odyssey infrared florescent scanner (LI-COR, United States) was used to detect the final protein separation. Purified Fis1 and Mid51 proteins were stored at −80 °C in affinity chromatography elution buffer or in SEC elution buffer. Next, the proteins were dialyzed into the proper buffer as needed in each protocol.

### 2.6 Peptide binding to protein, *in vitro*

In brief: We used the AGILE Dev Kit label-free binding assay (Cardea, San Diego, CA, United States) to determine peptide binding to immobilized proteins *in vitro*. Based on the manufacturer’s standard protocol (Lerner et al., 2022). Protein was immobilized/cross-linked into the carboxyl group present on the activated graphene biosensor chip. The analyte is applied in solution to the chip. As a result of an interaction, an alteration in the current (I) is measured and recorded continuously throughout the experiment. To determine the baseline equilibration response, phosphate-buffered saline (PBS) X 1 (pH 7.4) is used as a calibration solution. In order to perform the association step, analytes (50 μL) are diluted into PBS X 1 (pH 7.3). After the experiment, data were exported from three transistors and averaged, and any background drift recorded in PBS X 1 was subtracted.

Further details: All commercially available solvents and reagents were used without further purification. N-hydroxysulfosuccinimide sodium salt (Sulfo-NHS) was acquired from Biosynth Carbosynth (Compton, United Kingdom); 1-(3-Dimethylaminopropyl)-3-ethyl carbodiimide hydrochloride (EDC-HCl) was purchased from Alfa Aesar (Kandel, Germany); Quench 1 (3.9 mM amino-PEG5-alcohol in PBS (pH 7.4)) and Quench 2 (1 M ethanolamine (pH 8.5)) were purchased from Cardea (San Diego, CA, United States); 2-(N-morpholino)-ethane sulfonic acid (MES) was purchased from Sigma-Aldrich (Saint Louis, MO, United States); PBS X 10 (pH 7.0) was purchased from Hylabs (Rehovot, Israel).

Ethyl (dimethyl aminopropyl)carbodiimide and N-hydroxysuccinimide were used to link the capture molecules to the chip. The protein amine was covalently attached to the carboxyl on the chip using EDC (2 mg) and sulfo-NHS (6 mg) in MES buffer (1 M (pH 6.0)) for 15 min. A protein solution (500 nM) was incubated for 30 min on the chip. To quench the remaining unoccupied binding sites on the chip, Quench 1 was followed by Quench 2 for 15 min each. The baseline current levels for the chip were recorded for at least 5 minutes following a rinse in PBS. An aspiration of PBS was performed followed by the application of a droplet of the tested analyte to the sensor chip, followed by the recording of the change in the sensor chip readout, the aspiration of the analyte, and finally the rinsing of the chip with PBS. In order to calibrate, dissociate, regenerate, and rinse the system, a PBS X 1 buffer was used. Further measurements were conducted at varying analyte concentrations. Upon collecting data, the sensor responses on one assay chip were averaged and the background drift in PBS was subtracted. K_D_ was determined using a Hill fit plot. A statistical analysis program, GraphPad Prism 9, was used to calculate K_D_ values. Data presented as mean ± SD of all measurements. All samples were identical prior to treatment assignment.

### 2.7 *In vitro* competitive study

An AGILE Dev Kit (Cardea, San Diego, CA, United States) label-free binding assay was used to better visualize peptide inhibition activity. Using an AGILE Dev Kit label-free binding assay as described previously (Lerner et al., 2022; Sukumaran et al., 2023), we determined the interactions between the peptides and proteins by immobilizing proteins *in vitro*. The chips were immobilized with one protein, and a peptide and another protein were the analytes. At first, we bound the protein to the chip in the same manner as previously described (Lerner et al., 2022). Next, each analyte was diluted to a series of concentrations (10 points) to determine its K_D_. For calibration, dissociation, regeneration, and rinsing, PBS X 1 was applied. In the competitive study, the peptide concentration was constant, and different protein analyte concentrations were tested. One-hour incubation at 4 °C was performed on the protein and peptide. We calculated the K_D_ values using Nanomedical Agile Plus software, and we used Prism software to generate graphical representations and K_D_ values.

### 2.8 Pulldown assay

Protein-protein interaction assay was conducted using pulldown assay. Ni-Beads 200 μL (in 1.5 ml Eppendorf) were suspended and spun down. The supernatant was then discarded. Following the overnight suspension, the beads with wash buffer containing 5% Bovine serum albumin (BSA) were washed with BSA and transferred to a column. The column was washed three times with 200 µL wash buffer to remove all BSA from it. Next, 200 µL Mid51-His was incubated for 2 h, and the flow through (FT) was collected after washing. After that, 200 μL Fis1 (without His tag) 2 M ratio was incubated for 1 h. The collected FT, wash, and elution were subjected to Western blot.

### 2.9 Cell viability assay

The viability of H9c2 cells was measured using sodium 3-[1-(phenylaminocarbonyl)-3,4- tetrazolium]-bis (4-methoxy6-nitro) (XTT) benzene sulfonic acid hydrate assay, according to the manufacturer’s instructions and standard procedure (Kuznetsov et al., 2015). We cultivated H9c2 cells in Dulbecco’s Modified Eagle Medium (DMEM) containing fetal bovine serum (10% FBS), penicillin-streptomycin (100 U/mL–100 ug/mL), sodium pyruvate (1%), and sodium hydrogen carbonate (0.15%). A set of white 96-well plates with a clear bottom was seeded with H9c2 cells at a density of 1 × 10^4^ cells/well and incubated in a humidified atmosphere at 37°C with a supply of 5% CO_2_. Cells were incubated at 37°C with 5% CO_2_ and peptides (1 µM) for 14 h. Following this, XTT reagent (3.2 ml of XTT reagent and 64 µL XTT activator) was applied, and the results were analyzed after 6 hours. Cell viability analysis was conducted using the CellTiter-Glo Luminescent Cell Viability Assay kit by Promega (Madison, WI), as directed by the manufacturer. Cells were analyzed using TECAN Infinite M100 (NEOTEC, Scientific Instrumentation Ltd, Israel).

H9c2 cells obtained from embryonic rat heart tissue (CRL-1446) have been obtained from the American Type Culture Collection (ATCC, Gaithersburg, MD, United States); Biological Industries (Beit-Haemek, Israel) provided Dulbecco’s modified Eagle’s medium with high glucose, fetal bovine serum, sodium pyruvate, penicillin-streptomycin (10 X), combined antibiotic solutions, sodium hydrogen carbonate, sodium hydrogen carbonate, and penicillin-streptomycin (Pen-Strep).

### 2.10 Statistical analysis

The statistical analyses in this study were conducted using the statistical software GraphPad Prism 9.0. All data were presented as means by S.E.M and ±SD. Data from more than two groups were analyzed using one-way analysis of variance (ANOVA) followed by Tukey’s multiple comparison test. All experiments were replicated at least three times, except molecular docking results and *in silico* analysis calculations.

## 3 Results and discussion

### 3.1 Rational design of peptide inhibitor targeting Fis1/Mid51 PPI

**FIGURE 1:**
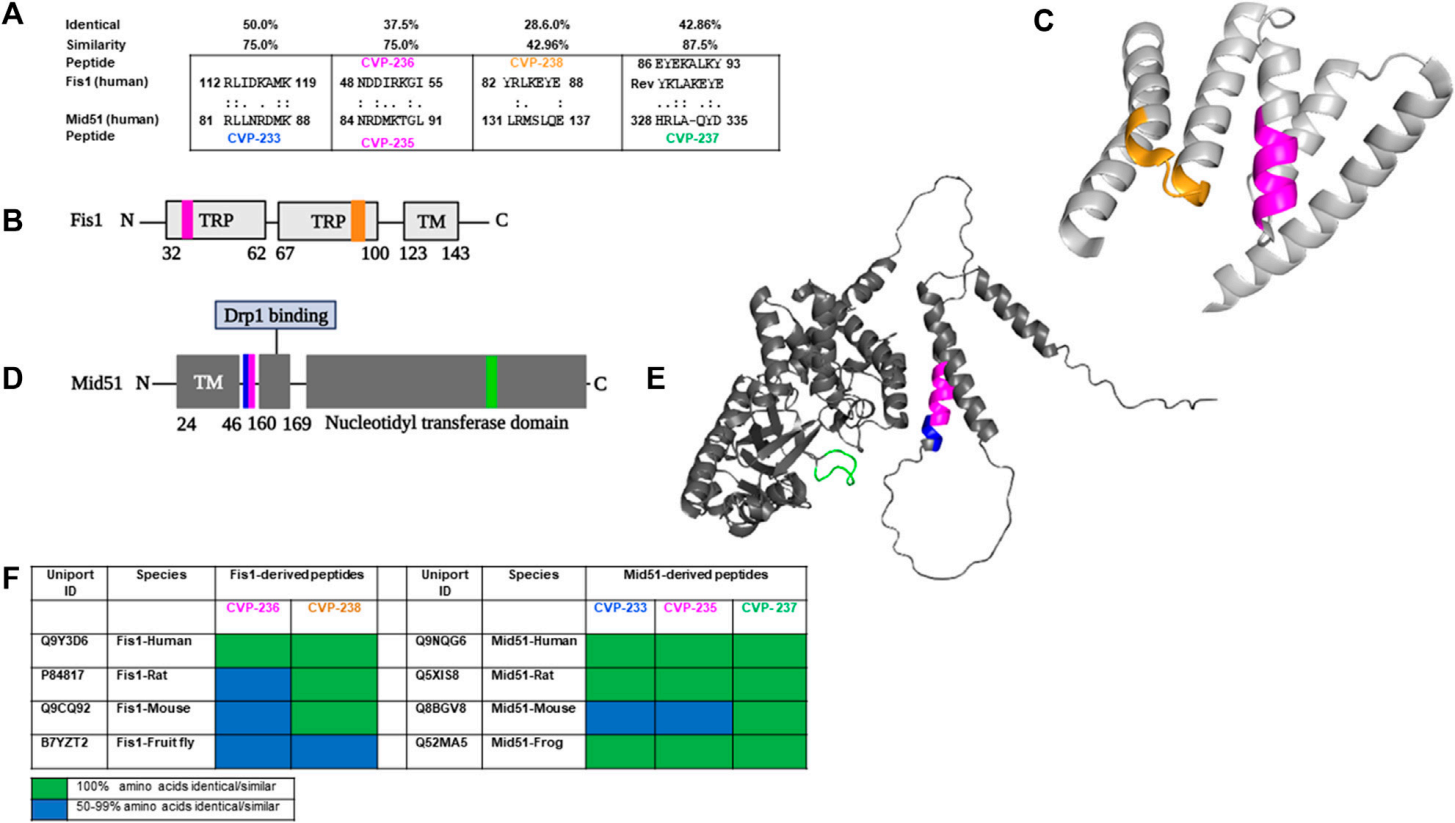
Rational design identifies peptides derived from Fis1/Mid51 PPIs. **(A)** Short sequences of homology between Fis1 and Mid51 representing CVP-233, CVP-235, CVP-236, CVP-237, and CVP-238 were identified using Lalign. ‘:’ and ‘.’ correspond to sequence complete identity and sequence similarity, respectively. **(B)** Schematic representation of Fis1 structural elements (152 AAs). Fis1 has two core tetratricopeptide repeat (TPR) structure motifs flanked by two helices (helices one and 6). The N-terminal region of Fis1 is called the “N-terminal arm”. The transmembrane domain (TM) structure in the membrane is unknown but presumably helical. Highlighted in the same colors are the two regions of homology between the two proteins, regions CVP-236 and CVP-238 in Fis1. **(C)** 3D structures of Fis1 protein is shown in colors that correspond to the respective peptide position sequence colors. Highlighted in the same colors are the two regions of homology between the two proteins, regions CVP-236 and CVP-238 in Fis1. AlphaFold prediction (AF-Q9Y3D6-F1 (Dohm et al., 2004)). **(D)** Schematic representation of Mid51 structural elements (463 AAs). Mid51 has an N-terminal transmembrane domain (TM), a Drp1 binding domain (Drp1 binding), and a nucleotidyl transferase domain (ADP binding). Highlighted in the same colors are the three regions of homology between the two proteins, regions CVP-233, CVP-235 and CVP-237 in Mid51. **(E)** 3D structure of Mid51 protein (AlphaFold:AF-Q9NQG6-F1 (Loson et al., 2015)). Highlighted in the same colors are the three regions of homology between the two proteins, regions CVP-233, CVP-235 and CVP-237 in Mid51. **(F)** Representing sequence conservation of human proteins and other species that contain a region identical/similar to the designed peptides. Sequence conservation corresponds to the number of residues identical/similar to the peptides in each sequence (for full sequence information see Supplementary Table S2). PyMol (Schrodinger LLC) was used to generate the figure (Schrödinger and DeLano, 2020).

It has been demonstrated that Fis1 interacts with Mid51 and recruits Drp1 independently to promote mitochondrial fission (Zhao et al., 2011; Losón et al., 2013). Activating mitochondrial fission and inhibiting mitochondrial fusion is one method Fis1 promotes mitochondrial fragmentation (Yu et al., 2019). Mid51, however, interacts with fusion proteins and leads to mitochondrial elongation rather than fission in many cells (Yu et al., 2020; Xian and Liou, 2021). Fis1, Mid51, Mfn1 and Mfn2 proteins appear to have a myriad of roles in mitochondrial dynamics and CVD. Fis1 promotes mitochondrial fragmentation both by activating fission and by inhibiting fusion (Yu et al., 2019). Therefore, inducing mitochondrial fragmentation leads to CVD. Besides the action in the fission process, Mid51 can competitively decrease the interaction of Fis1 with Mfn1 and Mfn2 (Yu et al., 2021). It was previously suggested that Fis1/Mid51 PPI regulation could provide a therapeutic target for CVD (Vásquez-Trincado and Schinkel, 2016; Liu et al., 2023). Furthermore, peptides corresponding to specific regions of interacting proteins have been shown to be effective PPI inhibitors (Disatnik et al., 2013; Qi et al., 2013). The hypothesis was that Fis1/Mid51 PPI inhibition would provide cardioprotection. In previous studies, P110 was identified as a specific Drp1/Fis1 inhibitor. The study demonstrated that Fis1 recruits Drp1 under stress in neurodegenerative disease models and that inhibiting Drp1/Fis1 PPI has a neuroprotective effect in these models (Qi et al., 2013). By using a rational design approach (Souroujon and Mochly-Rosen, 1998; Qvit et al., 2016a; Qvit et al., 2016b), we developed peptides that are homologous in amino acid sequence between Fis1 and Mid51 (Figure 1A). Multiple species share these domains, and only conservative amino acid substitutions were found in Mid51 and Fis1 (Figure 1F). These sequences are also unique (not present in other proteins, data not shown), and are exposed in Fis1 and Mid51 (Figure 1; Supplementary Table S1). Interestingly, in Fis1 protein the peptides are derived from the TRPs domains, which were demonstrated as common PPI domains (Egner et al., 2022).

### 3.2 Structural physicochemical characteristics of the peptides

The structural and biophysical properties of lead compounds play a significant role in determining the bioactivity and properties of drugs (Quemener et al., 2022). Our objective was, therefore, to analyze and characterize the structural properties of the designed peptides (Supplementary Figure S1) using several methods, including theoretical isoelectric point (pI), net charge, molecular hydrophobicity, topological polar surface area, number of atoms, and molar refractive index (Table 1).

**TABLE 1:** Physicochemical properties of the designed peptides.

Peptide name	Sequences	L	pI	NC	H (Kcal/mol)	TPSA (Å^2^)	N atoms	Molar refractivity
CVP-233	RLLNRDMK	8	12.13	+2	+15.64	497.23	72	190.77
CVP-235	NRDMKTGL	8	11.38	+1	+16.48	455.55	64	267.48
CVP-236	NDDIRKGI	8	7.33	0	+19.55	472.62	65	230.70
CVP-237	HRLAQYD	7	8.07	0	+14.99	429.12	64	225.74
CVP-238	YRLKEYE	7	7.10	0	+17.10	440.89	71	256.41

Abbreviations: L: length; pI: isoelectric point; NC: Net charge at physiological pH; H: hydrophobicity; TPSA: topological polar surface area; N atoms: number of atoms.

CVP-233 and CVP-235 are both basic and cationic (Table 1). According to their isoelectric points (12.13 and 11.38) and net charges (+2 and +1), respectively. These properties of the peptides are accounted for by their basic residues. In general, the structural properties prediction results (TPSA: 429–500 Å^2^, and Molar refractivity: 190–270) of all peptides fall within the range of the properties reported for FDA-approved peptide drugs (including both marketed drugs and clinical candidates) (Santos et al., 2016).

### 3.3 *In silico* evaluation of the pharmacokinetic and biological activity analysis

Using ADMETlab’s ‘Drug-likeness analysis’ and ‘ADME/T evaluation’ (Table 2), the drug-likeness of the peptides was predicted *in silico* (Dong et al., 2018). Lipinski’s rule of five (aka Pfizer’s rule of five or simply the rule of five (RO5)) (Lipinski et al., 1997) was formulated to aid in the development of oral bioavailable drugs. Lipinski’s rule of five is one of the preliminary criteria for evaluating the drug-like properties of ideal drug structures based on their physicochemical properties. It is generally considered that chemical molecules with LogP (the logarithm of the compound partition coefficient between n-octanol and water) ≤ 5, hydrogen bond donors (HBD) ≤ 5, hydrogen bond acceptors (HBA) ≤ 10, molecular weight (MW) ≤ 500 Da, and rotatable bonds (nRB) ≤ 10, are likely to have the chemical and physical properties to be orally bioavailable (Gorla et al., 2021). A compound with the characteristics listed above has the potential to be a drug candidate. As all peptides have LogP values less than 5, they have relatively ideal hexane water distribution coefficients. Interactions between peptides and targets will be affected by the number of hydrogen bonds formed between them. In the study none of the peptides adhered to the rule of five. However, many drugs have been demonstrated to violate the rule of five as well (Doak et al., 2014). To illustrate this point, two orally bioavailable cyclic peptides are explored, including cyclosporine A, which is a powerful immunosuppressant isolated from the fungus *Tolypocladim inflatum*, and MK-0616, an oral macrocyclic peptide inhibitor of proprotein convertase subtilisin/kexin type 9 (PCSK9), which increases the removal of low-density lipoprotein cholesterol (LDL-C) from the blood. Based on a phase 2b study, MK-0616 significantly reduced LDL-C levels in hypercholesterolemia patients. According to their chemical structure, cyclosporin A and MK-0616 fall beyond the rule of five space (Lipinski, 2016).

**TABLE 2:** Lipinski’s rule-of-five, water solubility, drug-likeness and medicinal chemistry friendliness of the peptides.

Parameters		Peptides						
		CVP-233	CVP-235	CVP-236	CVP-237	CVP-238	Cyclosporine A	MK-0616
Lipinski’s rule of 5	MW ≤ 500	1045.26	935.07	930.02	901.97	1000.11	1202.61	1616.00
	HBD ≤5	18	16	16	15	16	5	11
	HBA ≤10	16	16	17	15	17	12	20
	LogP ≤5	1.30	0.99	-0.10	-0.10	0.16	2.92	3.8
	nRB ≤10	47	41	41	35	40	15	35
Water solubility	Log S (ESOL)	1.85	3.10	2.85	-0.07	0.73	-8.15	-9.10
	Class (ESOL)	Highly soluble	Highly soluble	Highly soluble	Very soluble	Highly soluble	Poorly soluble	Poorly soluble
Drug-likeness	Bioavailability score	0.17	0.17	0.17	0.17	0.17	0.17	0.17
Medicinal chemistry	Lead likeness (number of violations)	No; 2	No; 2	No; 2	No; 2	No; 2	No; 2	No; 2
	Synthetic accessibility	9.01	8.05	7.88	7.35	7.85	10.00	11.20

The peptide sequences that were used for this analysis did not include the N-terminal acetyl. Abbreviations: MW, molecular weight; HBD, hydrogen bond donors; HBA, hydrogen bond acceptors; LogP - the logarithm of compound partition coefficient between *n-*octanol and water; nRB, rotatable bonds.

Based on topological polar surface area (TPSA) and the Lipinski rules, the bioavailability score provides an estimate of whether a peptide is orally bioavailable. We generated bioavailability radar plots using SwissADME and found that most peptides exhibit optimal lipophilicity, solubility, and saturation (Supplementary Figure S2).

The pharmacokinetic properties of drug candidates include their absorption, distribution, metabolism, excretion, and toxicity (ADMET). ADMET is a very useful tool when discovering new drugs, as a drug candidate that passes the ADMET analysis is less likely to fail in subsequent clinical trials (Han et al., 2019). As part of the standardized assessment, peptides were screened by ADMET analysis (Table 3). Based on random forest algorithms, gastrointestinal absorption (GIA) is estimated, and a value of 30% of GIA (%) is utilized as a criterion to differentiate between poor absorption (GIA (−)) and good absorption (GIA (+)) (Wang et al., 2017). All peptides showed high absorption (Table 3). In this manner, the peptides are predicted to be less permeable to absorption and will not be detected in the postprandial bloodstream.

**TABLE 3:** Predicted pharmacokinetics/ADMET (absorption, distribution, metabolism, excretion, toxicity) profile of the peptides as analyzed by using SwissADME.

Parameters		Peptides				
		CVP-233	CVP-235	CVP-236	CVP-237	CVP-238
Absorption	Water solubility	1.85	3.10	2.85	-0.07	0.73
	GIA (+/−)	+(low)	+(low)	+(low)	+(low)	+(low)
	Skin permeability (cm/s)	-14.58	-14.58	-14.58	-14.58	-14.58
	Pgp-substrate (Y/N)	Yes	Yes	Yes	Yes	Yes
Distribution	BBB (+/−)	BBB-	BBB-	BBB-	BBB-	BBB-
Metabolism	CYP2D6 inhibitor (Y/N)	No	No	No	No	No
	CYP3A4 inhibitor (Y/N)	No	No	No	No	No
	CYP1A2 inhibitor (Y/N)	No	No	No	No	No
Excretion	CYP2C19 inhibitor (Y/N)	No	No	No	No	No
	CYP2C9 inhibitor (Y/N)	No	No	No	No	No
Toxicity		Non-toxin	Non-toxin	Non-toxin	Non-toxin	Non-toxin

GIA, gastrointestinal absorption; Pgp, P-glycoproteinc substrate; BBB, blood-brain barrier; CYP2D6 inhibitor, cytochrome P450 2D6; CYP3A4, cytochrome P450 3A4; CYP1A2 inhibitor, cytochrome P450 1A2; CYP2C9 inhibitor, cytochrome P450 2C9.

One of the most critical properties of drugs that must function in the brain is their ability to cross the blood-brain barrier (BBB) (Wang et al., 2009). The BBB was estimated using support vector machine (SVM) algorithms and divided into two classes: BBB+ and BBB- (Dong et al., 2018). The peptides exhibited a negative BBB crossing potential (BBB-). The P-glycoprotein (Pgp), which is a cell membrane protein, is involved in the absorption, excretion, drug-drug interactions, and central nervous system (CNS) effects of drugs (Didziapetris et al., 2003). Through increased renal and biliary elimination, Pgp protects cells from potentially toxic compounds. As well, it has been suggested that it may limit the accumulation of drug molecules in the cytosol, thereby reducing their intestinal absorption and bioavailability (Wolking et al., 2015). Given that all peptides were Pgp-substrates, their intestinal absorption and bioavailability are likely to be low. The Cytochrome P450 (CYP) enzyme family plays a significant role in the development of drugs (Rostkowski et al., 2013). The human CYP3A4 isoform is one of the most important enzymes for drug metabolism, improving tissue and organism protection by synergistically removing drug molecules from P-glycoproteins (van Waterschoot and Schinkel, 2011). It was predicted that all peptides would have no inhibitory effect on CYP3A4. Following this, we further confirmed the non-toxicity of the peptides as predicted by ToxinPred (Table 3).

Pharmaceutical activity refers to the beneficial effects of drugs on living organisms. A bioactivity score analysis was conducted on the peptides for their potential activity against a variety of targets, including G-protein-coupled receptors (GPCRs), ion channel modulators, nuclear receptor ligands, and enzyme inhibitors (Othman et al., 2020). An active compound with a score greater than 0 is regarded as highly active, while a compound with a score between 0-(-0.5) is regarded as moderately active, and a compound with a score below (-0.5) is regarded as inactive (Murugesan et al., 2021). The peptides showed no activity against all targets (Table 4), yet it is recommended to further verify biological activity in experimental biological assays.

**TABLE 4:** Biological activity prediction of peptides.

Peptides	Parameters of bioactivity score					
	GPCR ligand	Ion channel modulator	Kinase inhibitor	Nuclear receptor ligand	Protease inhibitor	Enzyme inhibitor
CVP-233	-3.57	-3.73	-3.77	-3.78	-3.12	-3.64
CVP-235	-2.76	-3.54	-3.59	-3,58	-1.89	-3.09
CVP-236	-2.94	-3.57	-3.58	-3.66	-2.18	-3.27
CVP-237	-2.62	-3.42	-3.46	-3.63	-1.90	-3.03
CVP-238	-3.50	-3.68	-3.72	-3.74	-3.03	-3.61

### 3.4 Peptide synthesis and characterization

The peptides were synthesized with Rink amide-MBHA resin by Fmoc-based solid-phase peptide synthesis (SPPS) according to the standard protocol (for full information, see Materials and Methods). All peptides were characterized by MALDI-MS and HPLC (Supplementary Figure S3, 4; Supplementary Table S2).

### 3.5 Predict peptide docking

Binding to the target protein is one of the most crucial considerations in PPI modulator design. We used the HPEPDOCK2.0 web server (Zhou et al., 2018) to investigate the binding conformations, orientations, and binding modes of the peptide to the target proteins. Also, the peptide’s secondary structure was investigated using Chou and Fasman secondary structure prediction (Kumar, 2013). The docking server compares different peptide conformations using a hierarchical flexible peptide docking algorithm as a scoring function (Zhou et al., 2018). We performed a global search around the entire protein to determine the peptide’s putative binding orientation and chose the best peptide conformation (docking score in Table 5) from the ten best poses generated by HPEPDOCK2.0. HPEP docking allows for peptide flexibility without lengthy simulations. The peptides can be accommodated in the binding pocket formed upon binding to the target proteins. This pocket is stabilized by hydrogen bonds calculated with a cutoff value of< 3.4 Å. We predicted the docking score values of the peptides using HPEPDOCK (Table 5).

**TABLE 5:** Docking score of peptides to Fis1 (AlphaFold: AF-Q9Y3D6-F1) or Mid51 (AlphaFold: AF-Q9NQG6-F1) calculated by HPEPDOCK molecular docking.

Peptide name	Peptide derived	Binding target protein	Docking scores
CVP-233	Mid51	Fis1	-155.87
CVP-235	Mid51	Fis1	-150.26
CVP-236	Fis1	Mid51	-133.24
CVP-237	Mid51	Fis1	-173.33
CVP-238	Fis1	Mid51	-179.45

The highest docking score was -179.45 for peptide CVP-238, followed by -173.33, -155.87, -150.26 and -133.24 for CVP-237, CVP-233, CVP-235 and CVP-236, respectively (Table 5).

### 3.6 *In vitro* peptides binding

**FIGURE 2:**
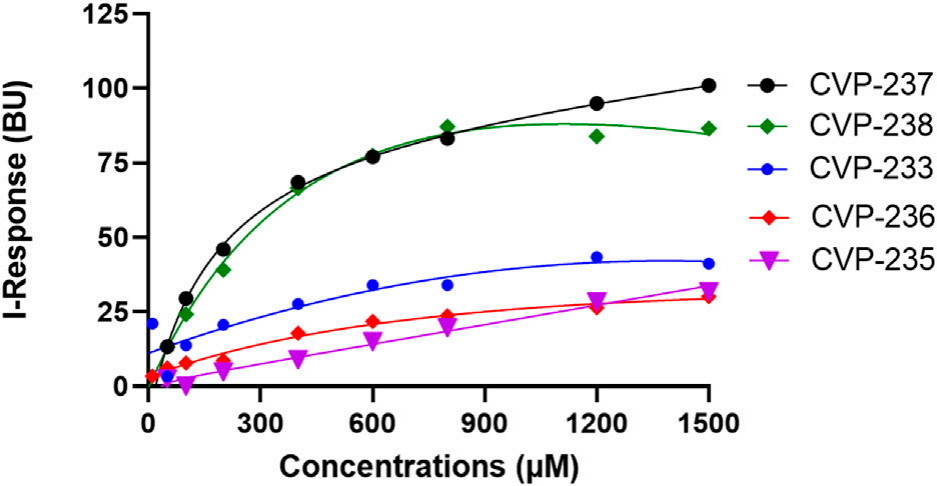
Binding interactions between peptides and target proteins, Fis1 and Mid51. Binding curves were generated using field-effect biosensing (FEB) technology. Protein (500 nM) was immobilized/cross-linked into the carboxyl group present on the activated graphene biosensor chip. The analyte/peptide is applied in a serious of concentration solution to the chip. The entire cycle of the experiment starts with calibration using PBS (pH 7.4) to record the baseline equilibration response at room temperature. The *Y*-axis corresponds to the I-Response in biosensor units (BU), and the *X*-axis corresponds to the different concentrations of the analyte peptides in the experiments. All experiments were performed at the same concentrations (n = 3).

Next, we examined the binding affinity of the peptides using field-effect biosensing (FEB) technology (Lerner et al., 2022). In this assay, a concentration-dependent interaction was observed for all peptides (Figure 2; Table 6; Supplementary Figure S5).

**TABLE 6:** Summary of the corresponding *in vitro* K_D_ and binding free energy values of the proteins and peptides binding.

Peptide name	Peptide derived	Binding target protein	K_D_ (µM)	*R* ^2^
CVP-233	Mid51	Fis1	1489.00 ± 0.19	0.88
CVP-235	Mid51	Fis1	3885.00 ± 10.24	0.99
CVP-236	Fis1	Mid51	862.60 ± 0.10	0.95
CVP-237	Mid51	Fis1	131.40 ± 0.10	0.99
CVP-238	Fis1	Mid51	466.00 ± 0.02	0.99

Interestingly, the peptides with the highest affinity, CVP-237, and CVP-238 (*in vitro* experimental data), are also the peptides with the highest docking scores -179.45 for peptide CVP-238 and -173.33 for CVP-237 based on *in silico* prediction.

### 3.7 Molecular docking of peptides to Fis1 and Mid51 proteins

Following *in silico* binding predictions and *in vitro* binding assays, CVP-237, and CVP-238 were chosen for further study using molecular docking. This was to investigate their interactions at the molecular level. The binding free energy of the complexes was calculated using hydrophobic interaction, electrostatic bond interaction, and Van der Waals interaction energy of the complex. The server generates four sets of models based on the following scoring schemes: balanced (denoted as 00 in the server), electrostatics-favored, hydrophobicity-favored, and Van der Waals (VdW) + electrostatics (Elec). In the balanced set 00, the weighting coefficients are selected according to similar weights for the four different energy terms. The top interacting ligands in each class that showed a strong correlation with binding energy were chosen as docked poses. In the docked position, all ligands demonstrated stability with ΔG binding energy > -100 kcal mol^−1^ (Table 7).

**TABLE 7:** Binding free energy of peptides to Fis1 (AlphaFold: AF-Q9Y3D6-F1) and Mid51 (AlphaFold: AF-Q9NQG6-F1) and their interaction favored weights (hydrophobic interactions; hydrogen bonds; electrostatic bonds; unfavorable bonds) calculated by molecular docking in kcal mol^−1^.

Peptide	Target protein	Balanced (Kcal mol^−1^)	Electrostatic favored (kcal mol^−1^)	Hydrophobic favored (kcal mol^−1^)	VdW + Elec (Kcal mol^−1^)
CVP-237	Fis1	-575.8	-597.4	-817.8	-120.3
CVP-238	Mid51	-656.4	-717.2	-919.9	-176.3

The hydrophobic interaction, electrostatic bond interaction, and VdW interaction free energy of the peptides and proteins were analyzed to determine features and characteristics that might facilitate binding capabilities and binding surface area. In the predicted binding free energy, the higher negative value means a higher binding affinity (Table 7). The peptides showed binding in the balanced predicted model. CVP-238 showed superior binding affinity in the electrostatic favor, displayed better binding affinity in the hydrophobicity favored, and lower binding free energy in VdW + Elec interaction models. Furthermore, the calculated molecular docking binding free energy results supported those found in the *in vitro* protein-peptide binding study.

### 3.8 Prediction of inhibitory action based on docking results

We predicted the inhibition activity of the peptides against the target proteins using molecular docking (Table 8). The prediction binding free energy value for Fis1/Mid51 PPI was -1324.9 kcal mol^−1^, indicating a strong interaction between Fis1 and Mid51. To further validate the peptide inhibition activity of Fis1/Mid51 PPI, we calculated the binding free energy in the presence of peptide and found higher free energy than the PPI values.

**TABLE 8:** Predicted competitive inhibition activity of CVP-237, and CVP-238 peptides against Mid51 and Fis1 interactions.

PPI	Binding free energy (kcal mol^−1^)	Inhibition (%)
Mid51 and Fis1	-1324.9	
Mid51 and Fis1 + CVP-237	-741.3	44.05
Mid51 and Fis1 + CVP-238	-747.4	43.59

**FIGURE 3:**
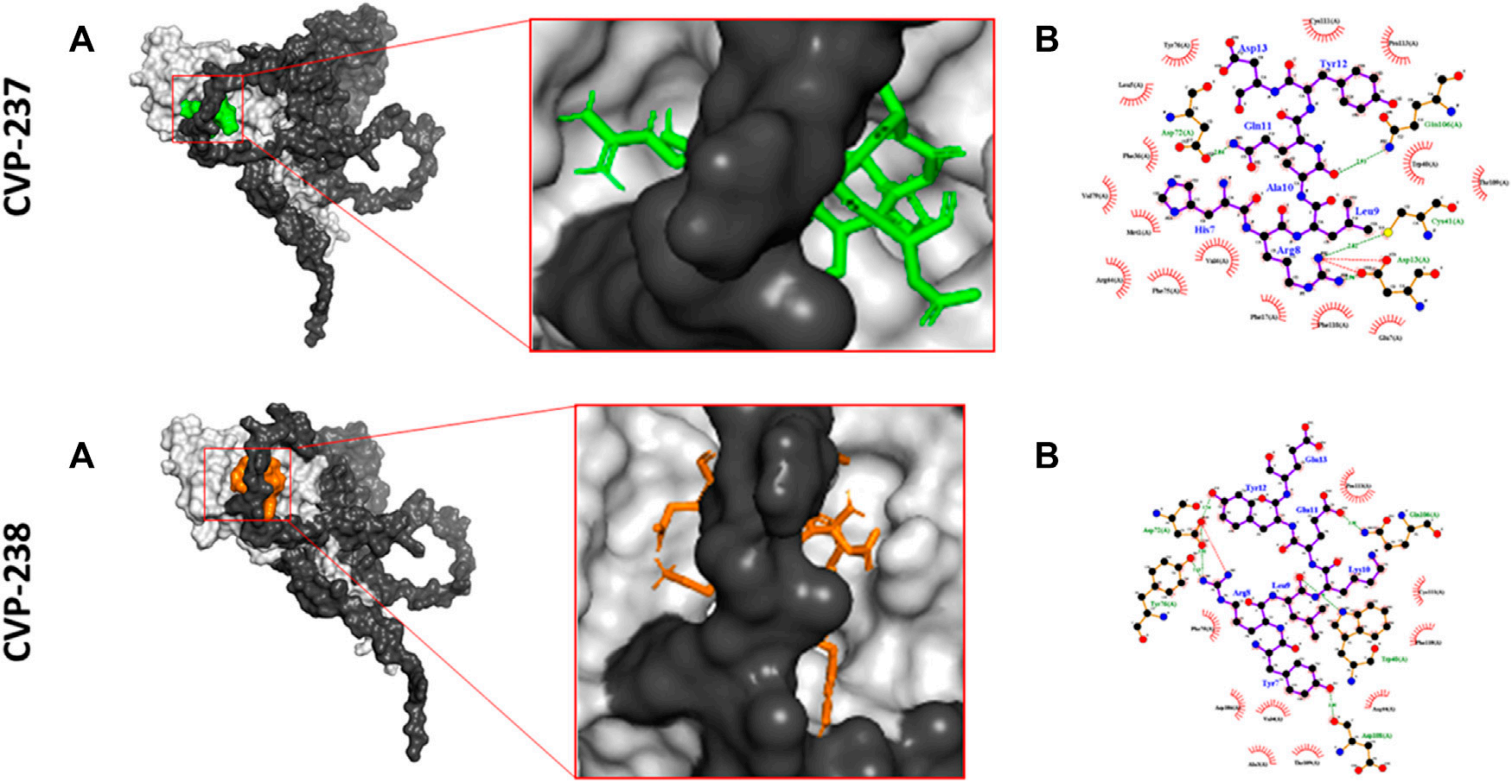
Predicted inhibition activity of peptides to Mid51 and Fis1 PPI docking model. In each peptide the docking model presented in 3D (left) indicated the peptides as cartons/sticks and 2D (right) diagrams in different colors and the receptor protein displayed as gray surfaces; **(A)** The cartoon representation of molecular docking inhibition prediction of the peptide between Mid51 and Fis1 is shown in the 3D diagram; **(B)** Protein bonds are shown in red in the 2D diagram, whereas peptide bonds are shown in purple. Hydrophobic bonds, external bonds, hydrogen bonds, and salt bridges are represented by red spoked arcs, purple lines, and green and red dashed lines. The projected view only shows the interacting residues at the binding interface (color figure created with LigPlot + v.2.2).

Molecular docking confirmed that the inhibition interaction domain and binding free energy of the proteins were compatible. The key predicted binding residues of Mid51 and Fis1 residues that are critical for binding complexes with the peptides are shown in 3D (Figures 3B, C; Figure 2D).

### 3.9 Design, chemistry peptide synthesis, and characterization

The two identified peptides were synthesized with a cell-penetrating peptide (CPP), *i.e.*, TAT carrier that enables safe and effective delivery of peptides into cells in culture, and *in vivo* (Schwarze et al., 1999; Begley et al., 2004) as well as a short spacer (Gly-Gly) for enhancing peptide flexibility. The peptides were synthesized by Fmoc-based solid-phase peptide synthesis (SPPS) using the standard procedure. The purity of the peptides was assessed using RP-HPLC and all the peptides used in biological assays were 100% pure (Supplementary Figure S3; Supplementary Table S2). We developed CVP-241, and CVP-242, which are composed of bioactive peptides (CVP-237 (HRLAQYD) and CVP-238 (YRLKEYE), respectively), a linker, and the CPP carrier. The peptides were cleaved from the resin, purified, and characterized by MALDI-MS and HPLC (Supplementary Figures S6, S7; Supplementary Table S3).

### 3.10 *In vitro* evaluation of peptide competitive activity

**FIGURE 4:**
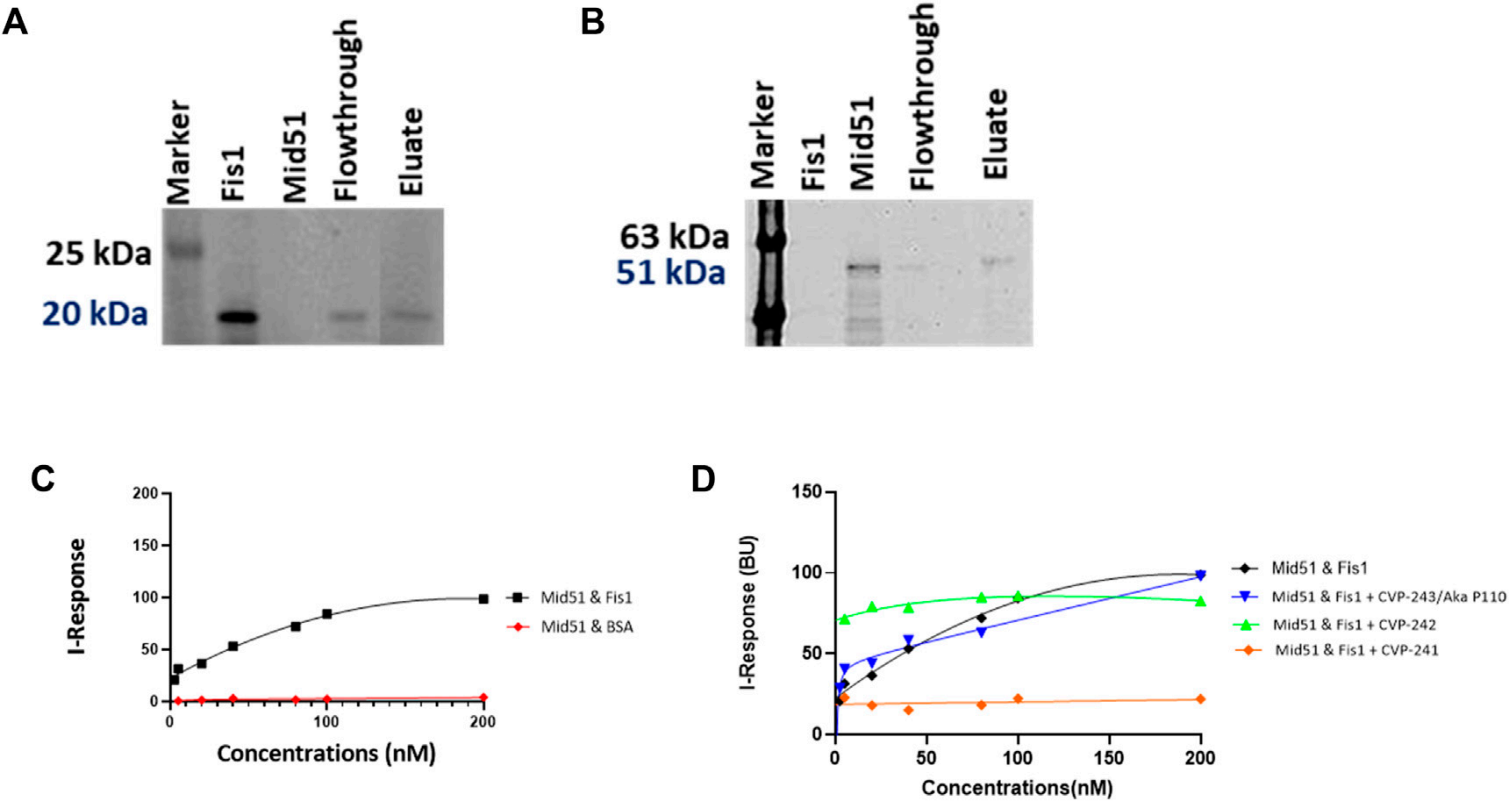
PPI and competitive inhibition studies *in vitro*. **(A, B)** Pull-down assays with Tag (His)-Tev-Mid51 (MW-51 kDa) and Fis1 (MW-20 kDa) using Ni-NTA beads with subsequent western blotting analysis. **(A)** Indicate the immunoblot with anti-Fis1 Primary antibody (SC-376447, Santa Cruz Biotechnology). **(B)** Indicate the immunoblot with anti-Mid51 (Primary antibody, SC-514135, Santa Cruz Biotechnology). **(C, D)** Binding curves for the PPI and competitive inhibition studies. The *Y*-axis corresponds to the I-Response in biosensor units (BU), and the *X*-axis corresponds to the different concentrations of the analyte in the experiment. **(C)** It is evidenced that there is a high interaction between the two proteins, Fis1 and Mid51 (Mid51 was immobilized on the chip and Fis1 was the analyte). A control study was also performed in which the biomolecular interaction between Mid51 and a control protein, Bovine serum albumin (BSA) was elucidated. The control study using BSA as an analyte was done using an identical experimental setup. It is evidenced that there is no interaction between Mid51 and the control BSA analyte **(D)** Both peptides, CVP-241 and CVP-242, demonstrate to reduce the interaction between the proteins. In addition, we tested CVP-243 (aka P110), a specific Drp1/Fis1 PPI inhibitor, which did not inhibit Fis1/Mid51 PPI (n = 3).

We evaluated Fis1/Mid51 through two different assays. Initially, a pulldown assay was used to characterize Fis1/Mid51 PPI. The results show that the two proteins interact (Figures 4A, B). Next, we used the FEB assay to further validate the Fis1/Mid51 PPI. The K_D_ value for Fis1/Mid51 interaction was found to be 0.054 µM, indicating a strong interaction between the target proteins. Finally, we studied competitive inhibition activity to verify the blockade effect of the peptides on Fis1/Mid51 PPI. Mid51 was immobilized and Fis1 was an analyte with a constant concentration of CVP-241. Also, Fis1 was immobilized and Mid51 was an analyte with a constant concentration of CVP-242. The competitive study results show a constant I-response when the analyte concentration increases in the presence of the peptide (Figure 4; Table 9; Supplementary Figures S8, S9). This suggests that these peptides block Fis1/Mid51 PPI. This data is supported by predicted molecular docking (Figure 3; Table 8). We also tested the inhibitory effect of CVP-243 (aka P110), a specific Drp1/Fis1 PPI inhibitor. As expected CVP-243 did not inhibit Fis1/Mid51 PPI.

**TABLE 9:** Competitive inhibition activity of CVP-241, CVP-242 and CVP-243 peptides within Fis1/Mid51 PPI.

Competitive study	K_D_ (µM)	*R* ^2^ value
Mid51 and Fis1	0.0539 ± 0.01	0.99
Mid51 and BSA	Unstable	NA
Mid51 and Fis1 + CVP-241	3.428 ± 0.05	0.96
Mid51 and Fis1 + CVP-242	44.579 ± 4.53	0.95
Mid51 and Fis1 + CVP-243	0.0570 ± 0.03	0.98

### 3.11 Cell viability study

**FIGURE 5:**
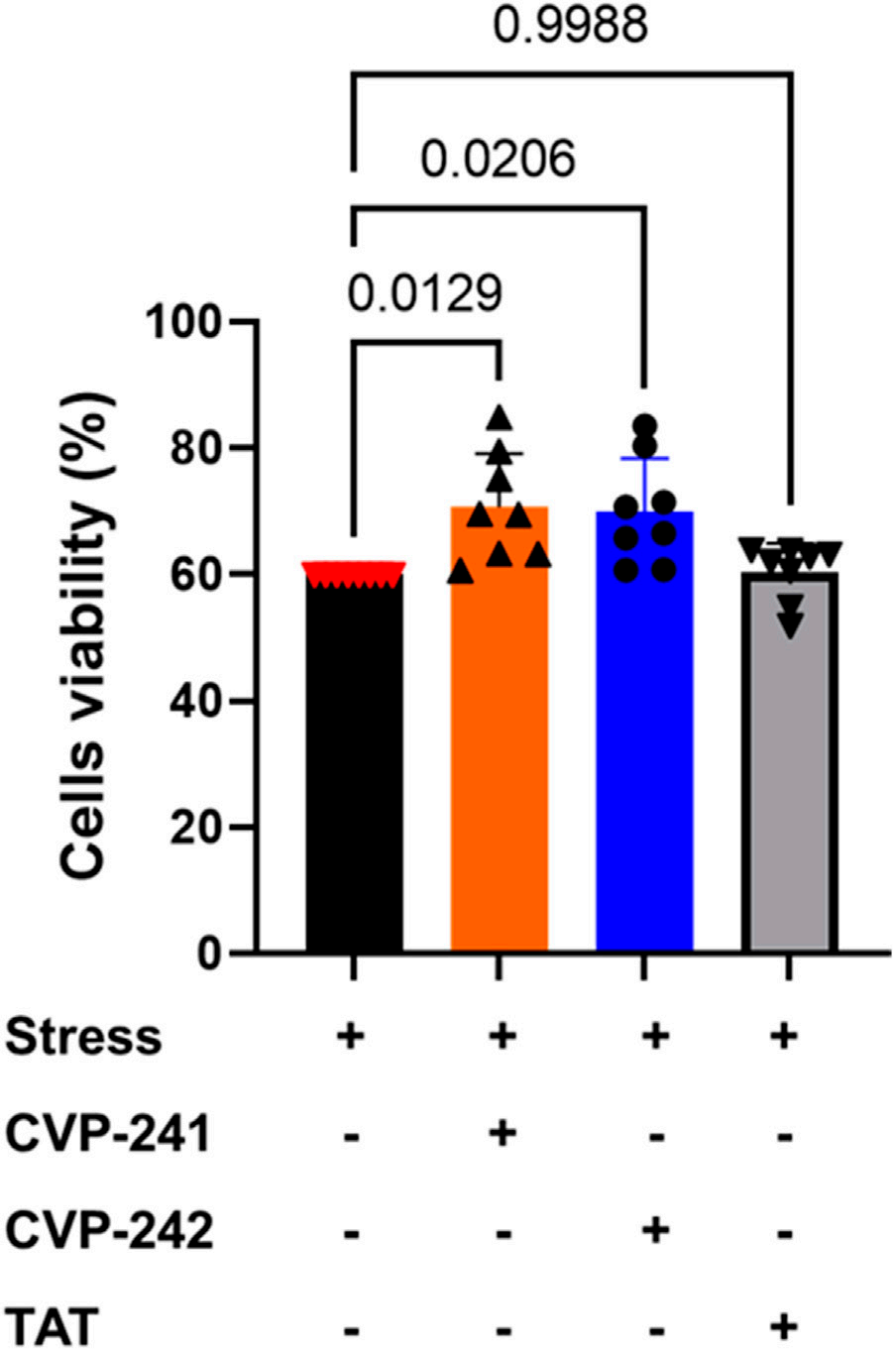
In cultured cardiomyocytes, peptide treatment reduced cell death induced by mitochondrial stressors. H9c2 cardiomyocytes, 4 × 10^3^ cells, were seeded in 96-well plates for 24 h, then incubated with Cobalt (II) (500 μM) and peptides, CVP-241, CVP-242, or TAT (negative control) (1 µM). Cell viability was measured by XTT release. Data are means ± SEM. One-way variance analysis (ANOVA) with *post hoc* Duncan analysis (n = 8).

We then investigated the bioactivity of the peptides in cardiomyocytes, H9c2 cells, under hypoxic conditions using colorimetric XTT assays. The peptides (CVP-241, and CVP-242) increased cell viability (Figure 5; Supplementary Table S4).

### 3.12 Peptides toxicity

**FIGURE 6:**
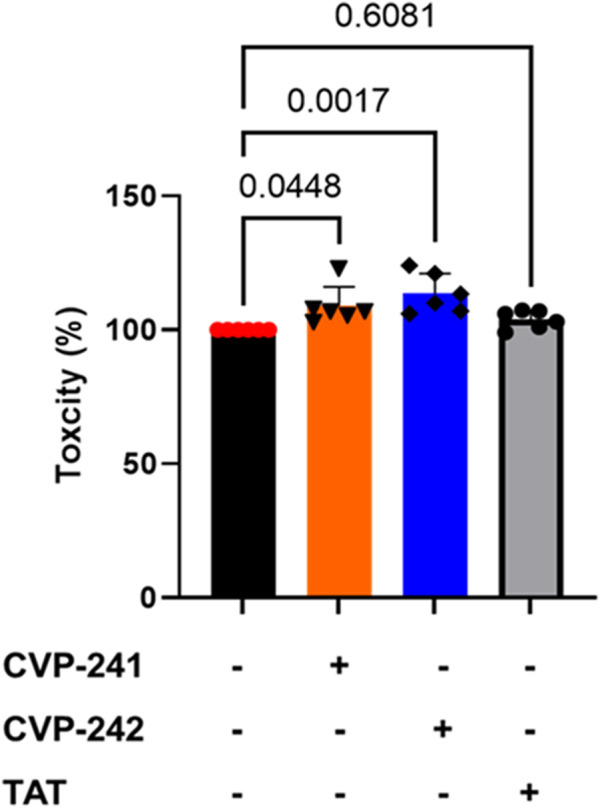
Toxicity effects of peptide treatment on cultured cardiomyocytes. We tested the peptide’s cell toxicity using H9c2 cardiomyocytes. 4 × 10^3^ cells were seeded in 96-well plates for 24 h, then incubated with peptide (1 μM) (CVP-241, CVP-242, or TAT) for 14 h. Data are means ± SEM. One-way analysis of variance (ANOVA) with *post hoc* testing by Duncan analysis (n = 6).

To examine peptide toxicity, an XTT assay was performed on cardiomyocytes. Peptide treatment did not reduce cell viability compared to non-treatment (NT). It can be seen that the peptides are non-toxic (Figure 6). These results agree with the predicted toxicity study used to predict the toxicity of the peptide, in which none of the peptides was toxic (Table 3).

## 4 Conclusion

In this study, we developed novel peptides that could serve as PPI inhibitors for Fis1/Mid51 PPI. In the first step, we developed novel peptides that could serve as potential inhibitors of Fis1/Mid51 PPI by using the rational design approach. Next, based on *in silico*, *in vitro*, and molecular docking we predicted the molecular interactions between the peptides and target proteins. Finally, we evaluated the peptide’s bioactivity *in vitro* and in cells. Two peptides inhibited Fis1/Mid51 *in vitro* as well as increased cell viability in cardiomyocytes in an ischemia-reperfusion model. Moreover, the peptides did not demonstrate toxicity in both models, *in silico*, and in cells.

While designing the peptides we noticed that there is an overlap of four amino acids between CVP-233 and CVP-235 and they represent similar properties regarding predicting docking scores (Table 5). Yet the net charge at physiological pH of CVP-233 is +2, while CVP-235 is +1. Interestingly, CVP-233 binding affinity to the target protein, Fis1, is significantly higher (better) (K_D_ ∼ 1489.00 µM) than CVP-235 (K_D_ ∼ 3885.00 µM). However, the most effective peptide binders had no charge at physiological pH at all.

Based on the initial design peptide CVP-237 is the most conserved peptide in evolution and CVP-238 sequence is identical in mammals. Both peptides are also derived from loop domains of the corresponding proteins (Mid51 and Fis1, respectively) (Figure 1). CVP-238 (YRLKEYE, Fis1 amino acids 82–88) has a relatively low identity and similarity (29% and 43%, respectively) to the amino acid sequence it derived from in Mid51 (LRMSLQE, amino acids 131–137). Yet there were several indications that this peptide may derive from a domain important for Fis1/Mid51 PPI and therefore be bioactive. Both sequences are derived from helixes that are considered important for PPI in each protein. In addition, peptide CVP-237 (HRLAQYD, Mid51 amino acids 328–335) is derived from the Fis1 reverse sequence (YKLAKEYE), in which the native sequence is EYEKALKY (Fis1 amino acids 86–93) and demonstrates significant overlap with CVP-238, YRLKEYE. Likewise, even amino acids that do not overlap have similar properties. While EYE overlaps between both sequences. The other amino acids in both peptides include aromatic, positively charged, and aliphatic amino acids (YRL vs YKL).

Moreover, both peptides represent some important critical ‘Drug-likeness’ physicochemical properties, such as low topological polar surface area (TPSA) (CVP-237–429 Å^2^, and CVP-238–441 Å^2^) (Table 1), as well as promising predictions based on Lipinski’s rule of five (Table 2), pharmacokinetics/ADMET (Table 3), and biological activity (Table 4) analysis. In addition, both peptides demonstrate the best docking score (Table 5) binding affinity to target proteins *in silico* (Table 7) and *in vitro* (Figure 2; Table 6). Finally, the peptides inhibited Fis1/Mid51 PPI based on *in silico* calculation (Table 8). And, it is clear from the prediction of inhibitory action based on the docking results, that both peptides have several common key binding residues involved in the complex, such as Trp40, Arg44, Phe75, Tyr76, Gln106, Thr109, Cys111, and Pro113 (Figure 3).

Next, we synthesize the peptides with the TAT sequence to deliver them to cells. While previous calculations were done on peptides without the TAT sequence, we believe that these analyses are critical to evaluating the cargo’s ‘drug likeness’ and binding properties. Indeed, both peptides (with TAT sequence) inhibited Fis1/Mid51 PPI (Table 9; Figure 4) and were shown to be cardioprotective in a cardiomyocyte viability assay (Figure 5), as well as no toxicity in H9c2 (Figure 6).

Mitochondria are highly plastic and dynamic organelles critical for mitochondrial metabolism, integrity, and homeostasis, and alterations in mitochondrial dynamics, such as fission and fusion, underlie various human diseases, including cancer, neurologic and CVDs. While initially Mid51 was characterized as a mitochondrial receptor for Drp1 recruitment to mitochondria to induce mitochondrial fission. Nevertheless, Mid51 overexpression in many cells leads to mitochondrial elongation rather than mitochondrial fission. Therefore, whether Mid51 acts as a positive or negative regulator of mitochondrial homeostasis is less clear. Fis1 was the first adaptor identified for Drp1 on the OMM and demonstrated to play a critical role in mitochondrial fission. However, several recent studies have confirmed the role of Fis1 in fusion. In addition, there are some indications for Fis1/Mid51 PPI which may imply that there is also crosstalk between fission and fusion machineries. Here we demonstrate Fis1/Mfn1 PPI and develop pharmacological tools to regulate it, to further reveal their roles in mitochondrial homeostasis and cell fate.

## Data Availability

The raw data supporting the conclusion of this article will be made available by the authors, without undue reservation.
