# Supplementary material for: Selective inhibitors targeting Fis1/Mid51 protein-protein interactions protect against hypoxia-induced damage in cardiomyocytes
Source: Front Pharmacol. 2023 Dec 21;14:1275370. doi: 10.3389/fphar.2023.1275370 (PMC10773907; doi:10.3389/fphar.2023.1275370)
Supplement: Supplementary file 1 [file DataSheet1.docx]

**Supplementary Information**

**Supplementary Table 1.** Rational peptide design identifies peptides derived from Fis1/Mid51 PPIs. Representing sequence conservation of human proteins and other species that contain a region identical/similar to the designed peptides. Sequence conservation corresponds to the number of residues identical/similar to the peptides in each sequence.

| **Uniport ID** | **Species** | **Fis1-derived peptides** | |  | **Uniport ID** | **Species** | **Mid51-derived peptides** | | |
| --- | --- | --- | --- | --- | --- | --- | --- | --- | --- |
|  |  | **CVP-236** | **CVP-238** |  |  |  | **CVP-233** | **CVP-235** | **CVP- 237** |
| **Q9Y3D6** | **Fis1-Human** | **NDDIRKGI** | **YRLKEYE** |  | **Q9NQG6** | **Mid51-Human** | **RLLNRDMK** | **NRDMKTGL** | **HRLAQYD** |
| **P84817** | **Fis1-Rat** | **NDDIRRGI** | **YRLKEYE** |  | **Q5XIS8** | **Mid51-Rat** | **RLLNRDMK** | **NRDMKTGL** | **HRLAQYD** |
| **Q9CQ92** | **Fis1-Mouse** | **NEDIRRGI** | **YRLKEYE** |  | **Q8BGV8** | **Mid51-Mouse** | **RLLNKDMK** | **NKDMKAGL** | **HRLAQYD** |
| **B7YZT2** | **Fis1-Fruit fly** | **TNDVRKGI** | **ARIKEYT** |  | **Q52MA5** | **Mid51-Frog** | **RLLNRDMK** | **NRDMKTGL** | **HRLAQYD** |

**Supplementary Figure 1.** Structures of designed peptides.


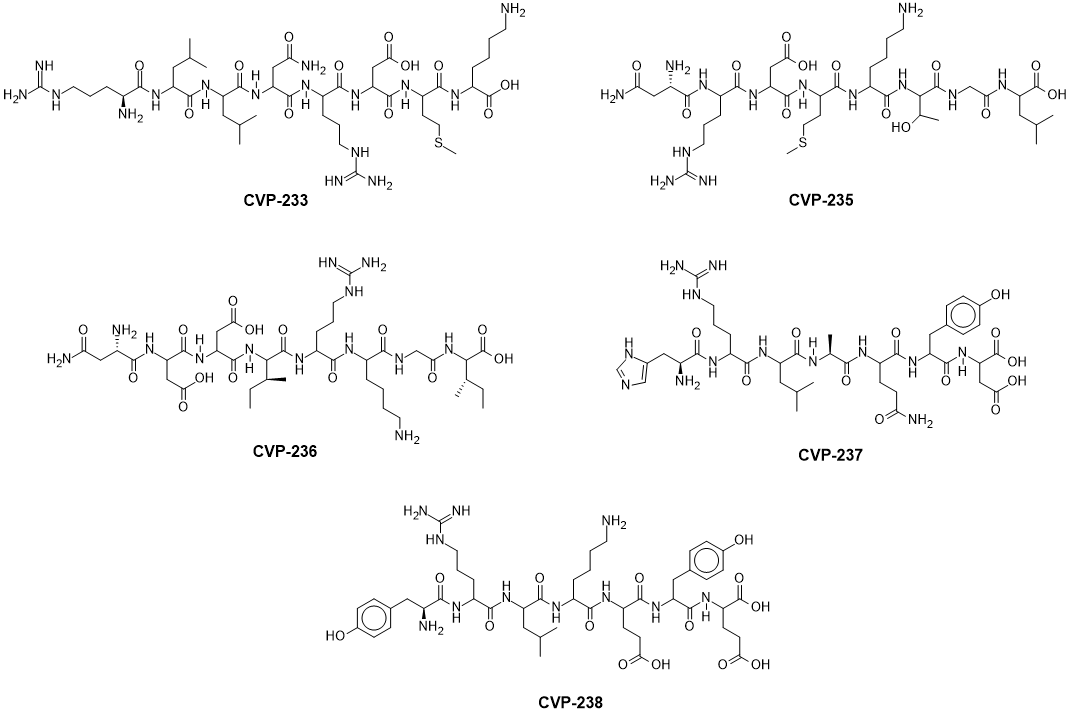


**Supplementary Figure 2.** Representative bioavailability radar plots of the peptides. The colored zone is the suitable physicochemical space for oral bioavailability. LIPO, lipophilicity; SIZE, molecular weight; POLAR, polarity; INSOLU, insolubility; INSATU, instauration; FLEX, flexibility. Peptides in dashed squares had moderate bioavailability scores (0.55).

CVP-233 CVP-235 CVP-236


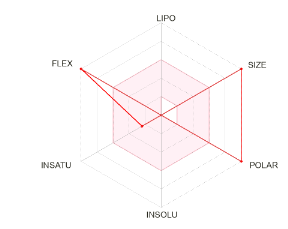

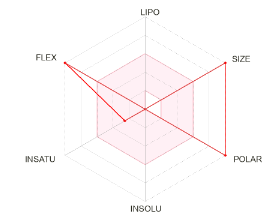

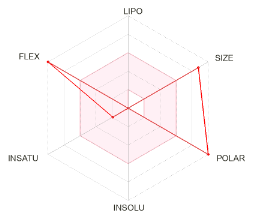


CVP-237 CVP-238


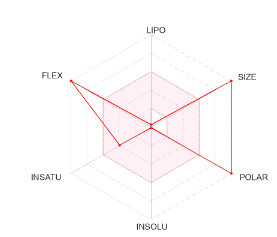

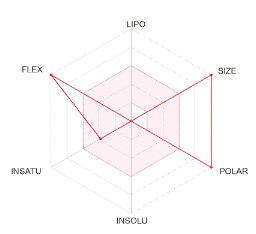


**Supplementary Figure 3.** Diagram of HPLC chromatography representing different peptide purity levels. (**A**) Chromatography diagram of CVP-233; (**B**) Chromatography diagram of CVP-235; (**C**) Chromatography diagram of CVP-236; (**D**) Chromatography diagram of CVP-237; (**E**) Chromatography diagram of CVP-238.

**A.**
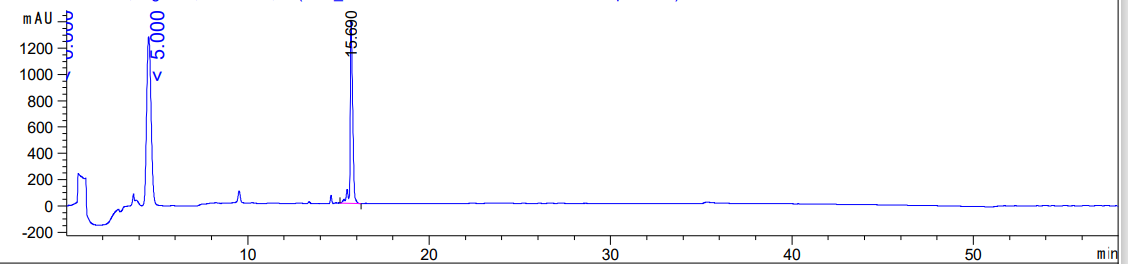


**B.**
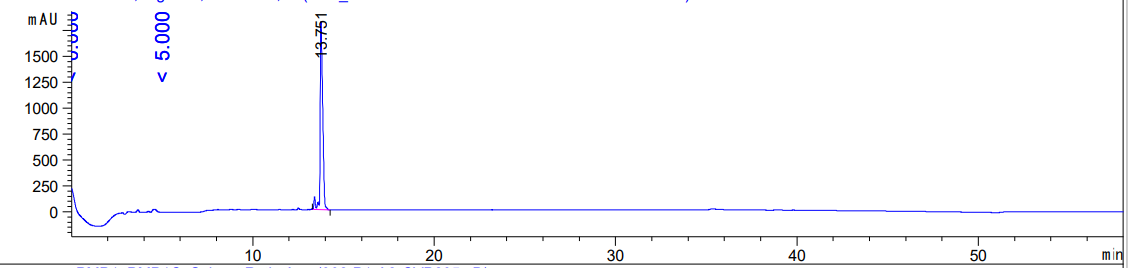


**C.**
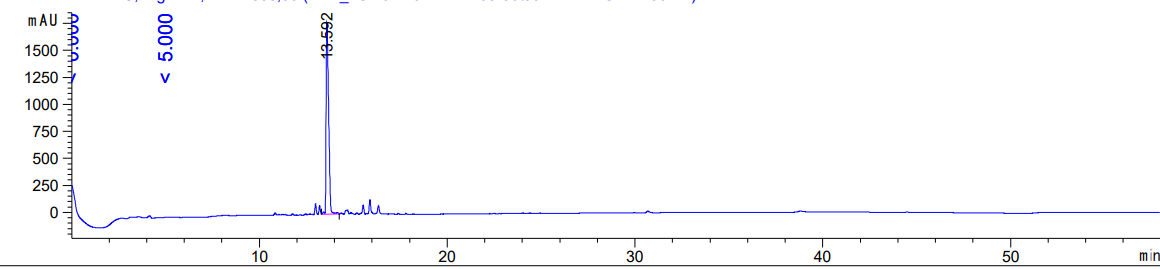


**D.**
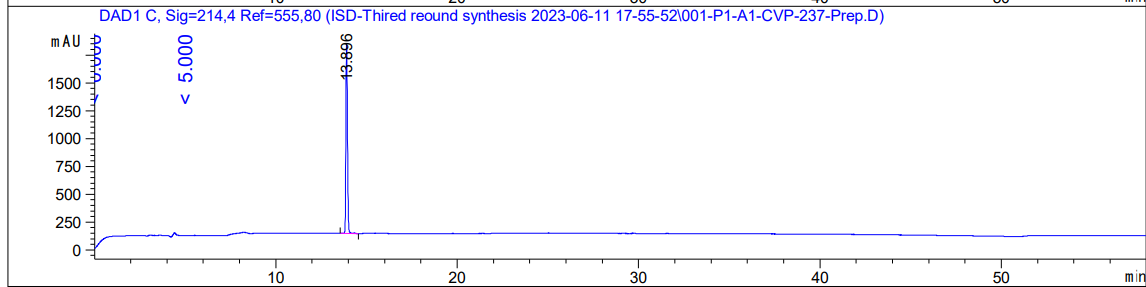


**E.**
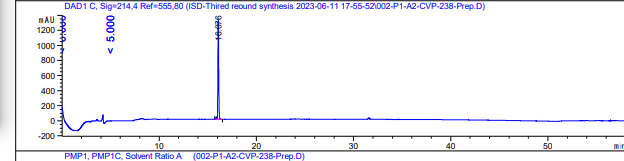


**Supplementary Figure 4.** Matrix-assisted laser desorption/ionization (MALDI) MS diagrams. (**A**) MS chromatography diagram of CVP-233; (**B**) MS chromatography diagram of CVP-235; (**C**) MS chromatography diagram of CVP-236; (**D**) MS chromatography diagram of CVP-237; (**E**) MS chromatography diagram of CVP-238.

**A.**
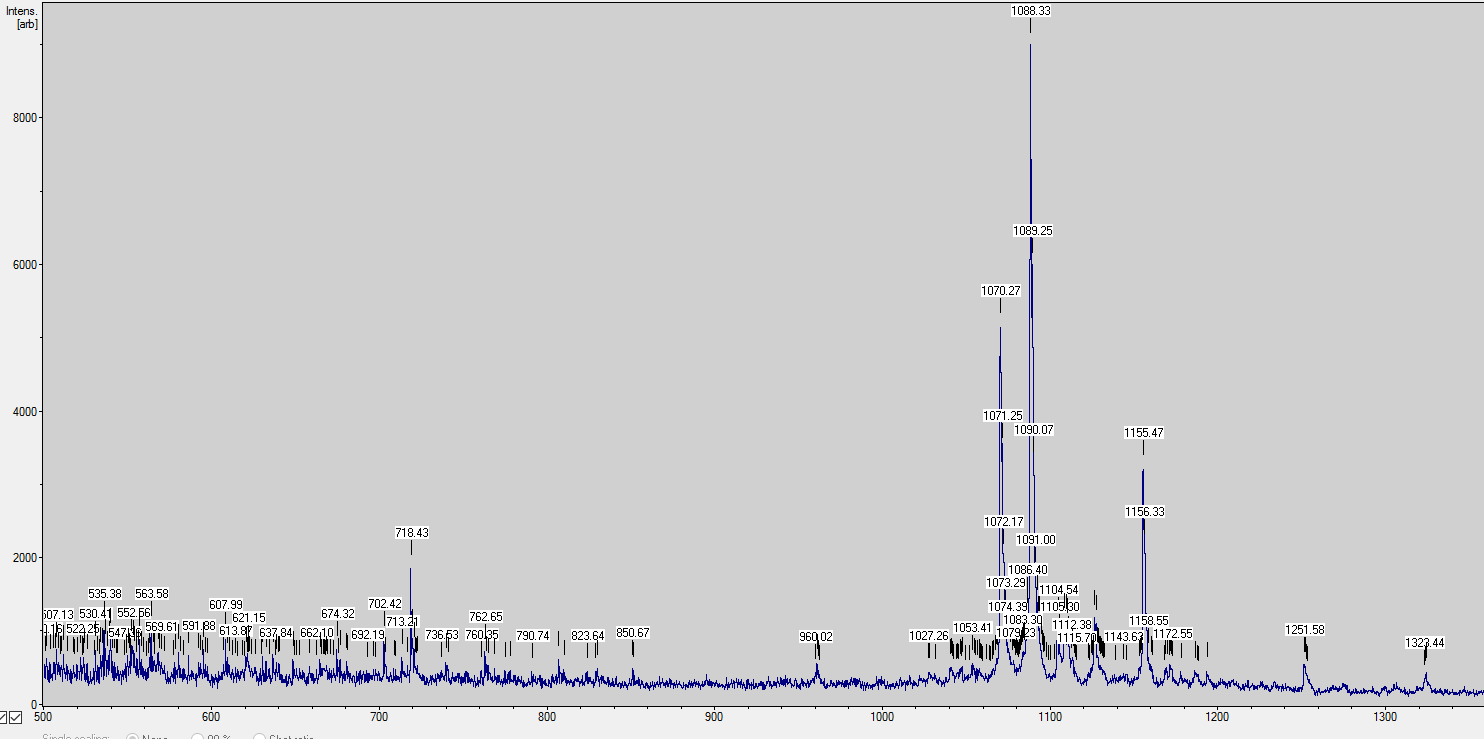


**B.**
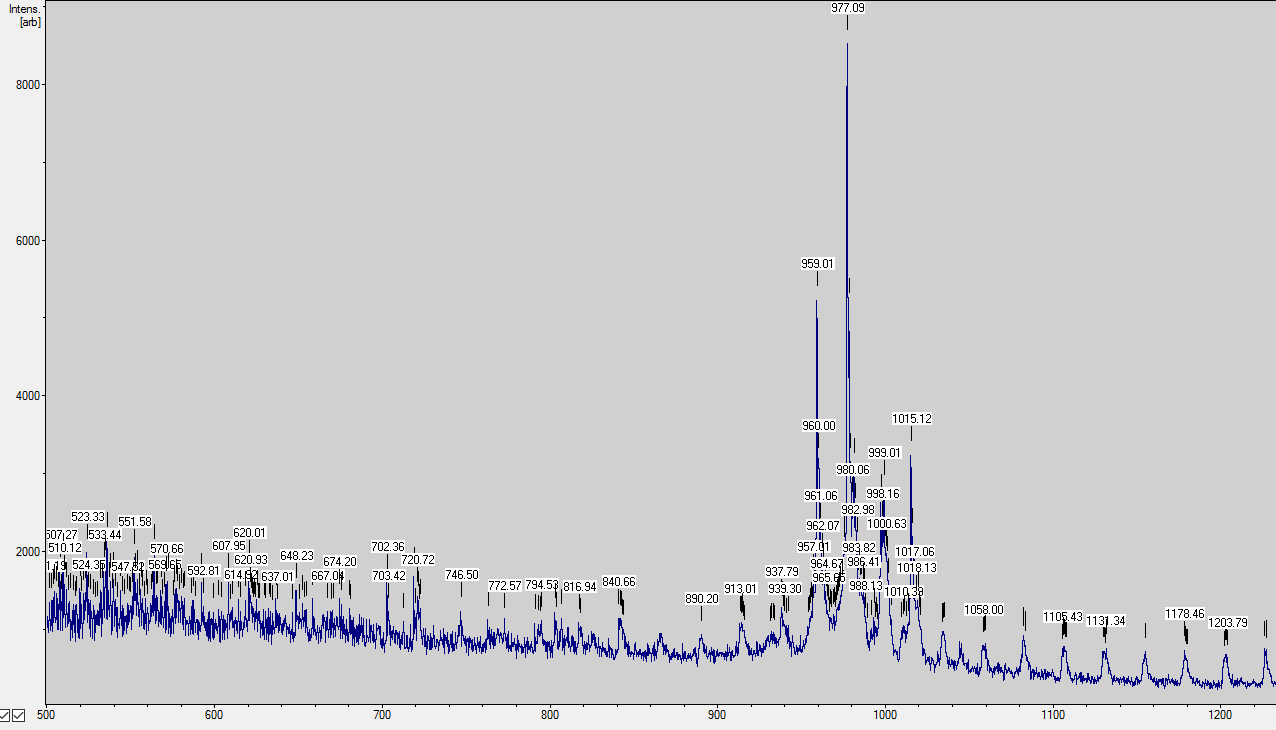


**C.**
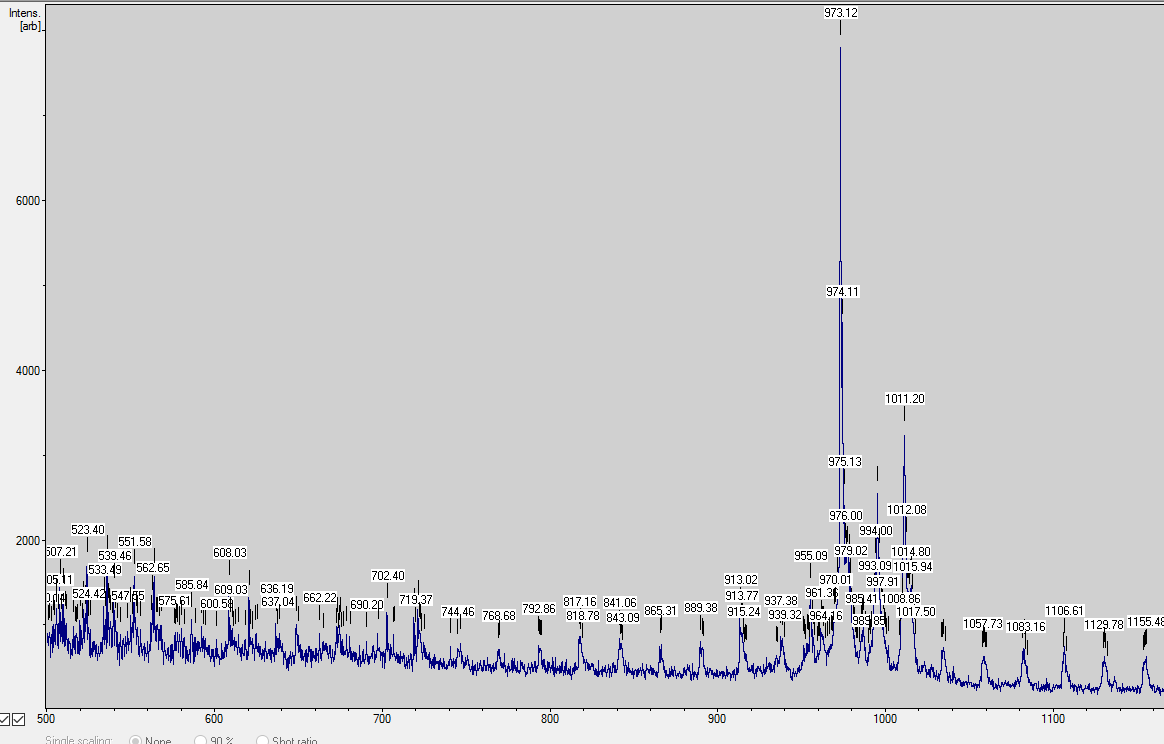


**D.**
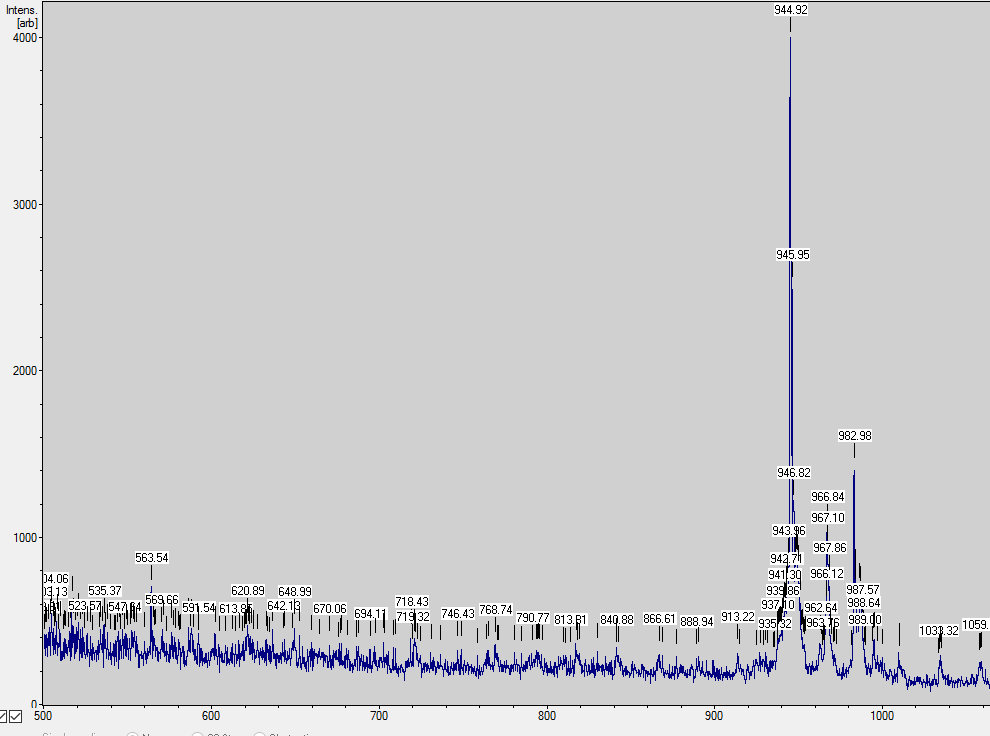


**E.**
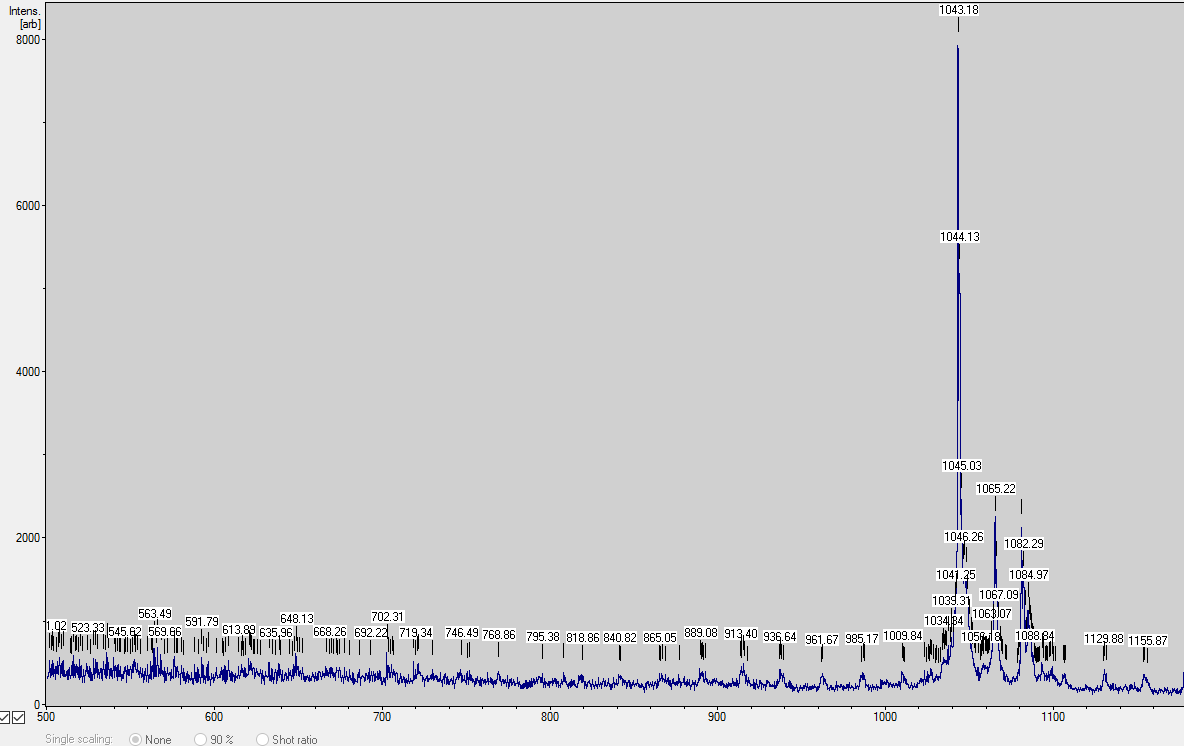


**Supplementary Table 2.** Characterization of the developed peptide library.

| **Peptide** | **Derived from** | **HPLC**  **(%)** | **MS Obs. (g/mol)** | **MS Cal. (g/mol)** |
| --- | --- | --- | --- | --- |
| CVP-233 | Mid51 | 100.00 | 1088.33 | 1087.30 |
| CVP-235 | Mid51 | 100.00 | 977.06 | 976.11 |
| CVP-236 | Fis1 | 100.00 | 973.12 | 972.05 |
| CVP-237 | Mid51 | 100.00 | 944.92 | 944.00 |
| CVP-238 | Fis1 | 100.00 | 1043.18 | 1042.14 |

**Supplementary Figure 5.** Fis1 and Mid51 (based on the target peptide) were immobilized on the chip (500 nM) and peptides (CVP-233, CVP-235, CVP-236, CVP-237, and CVP-238) were analyzed at 10 different concentrations (10 µM, 50 µM, 100 µM, 200 µM, 400 µM, 600 µM, 800 µM, 1,200 µM, 1,500 µM, and 2,000 µM). Field-effect biosensing (FEB) technology monitors experimental data in real-time. The Y-axis corresponds to the I-Response in biosensor units (BU), and the X-axis corresponds to the different time points and concentrations of the analyte in the experiment. (**A**) Graphical representation of Fis1 and CVP-233 peptide-protein binding experiment; (**B**) Graphical representation of Fis1 and CVP-235 peptide-protein binding experiment; (**C**) Graphical representation of Mid51 and CVP-236 peptide-protein binding experiment; (**D**) Graphical representation of Fis1 and CVP-237 peptide-protein binding experiment; (**E**) Graphical representation of Mid51 and CVP-238 peptide-protein binding experiment.

**A.**
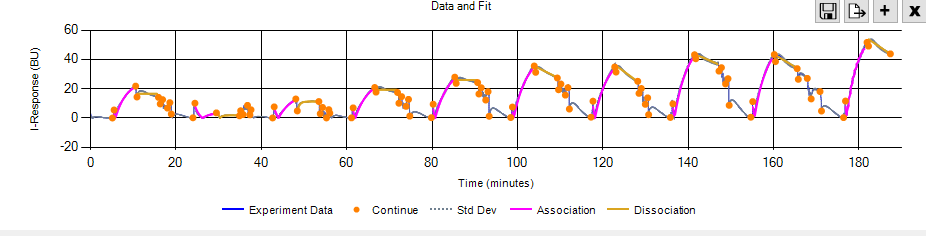
**B.**
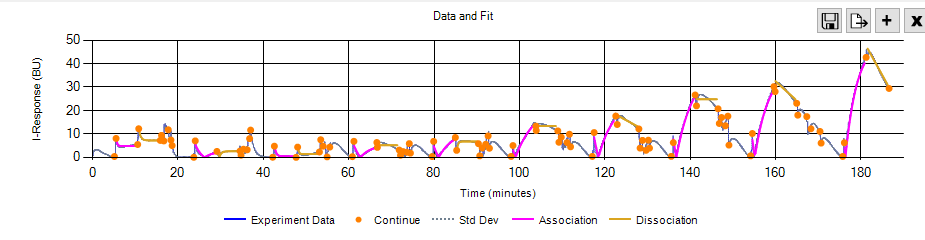


**C.**
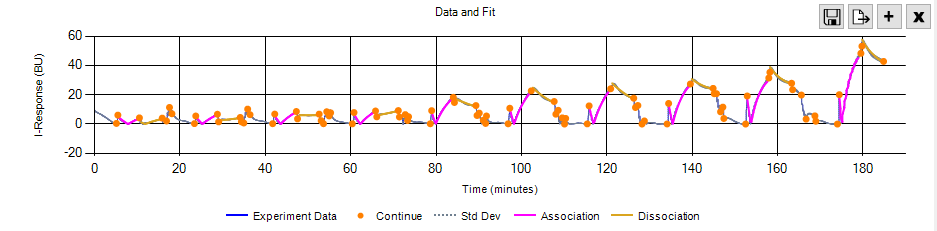


**D.**
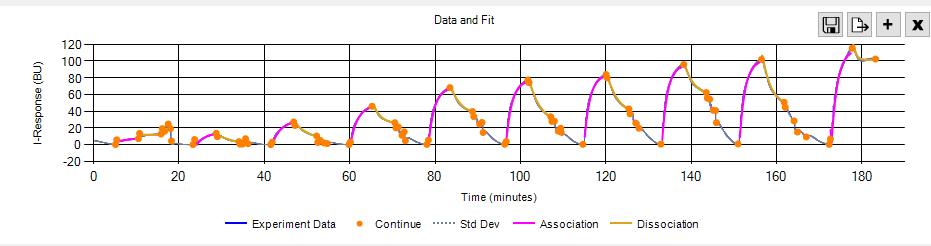


**E.**
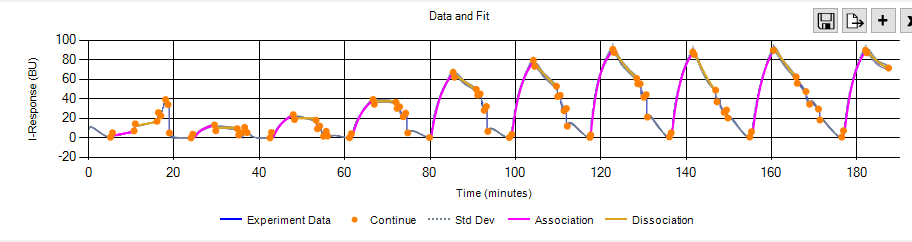


**Supplementary Figure 6**. Synthesis and characterization of CVP-241. (**A**) Structure of the peptide CVP-241. The bioactive peptide cargo (blue), and a short Gly-Gly spacer (red) conjugated with the TAT sequence (black) are highlighted with different colors. (**B**) HPLC Chromatography diagram of CVP-241. (**C**) Matrix-assisted laser desorption/ionization (MALDI) of CVP-241.

1.
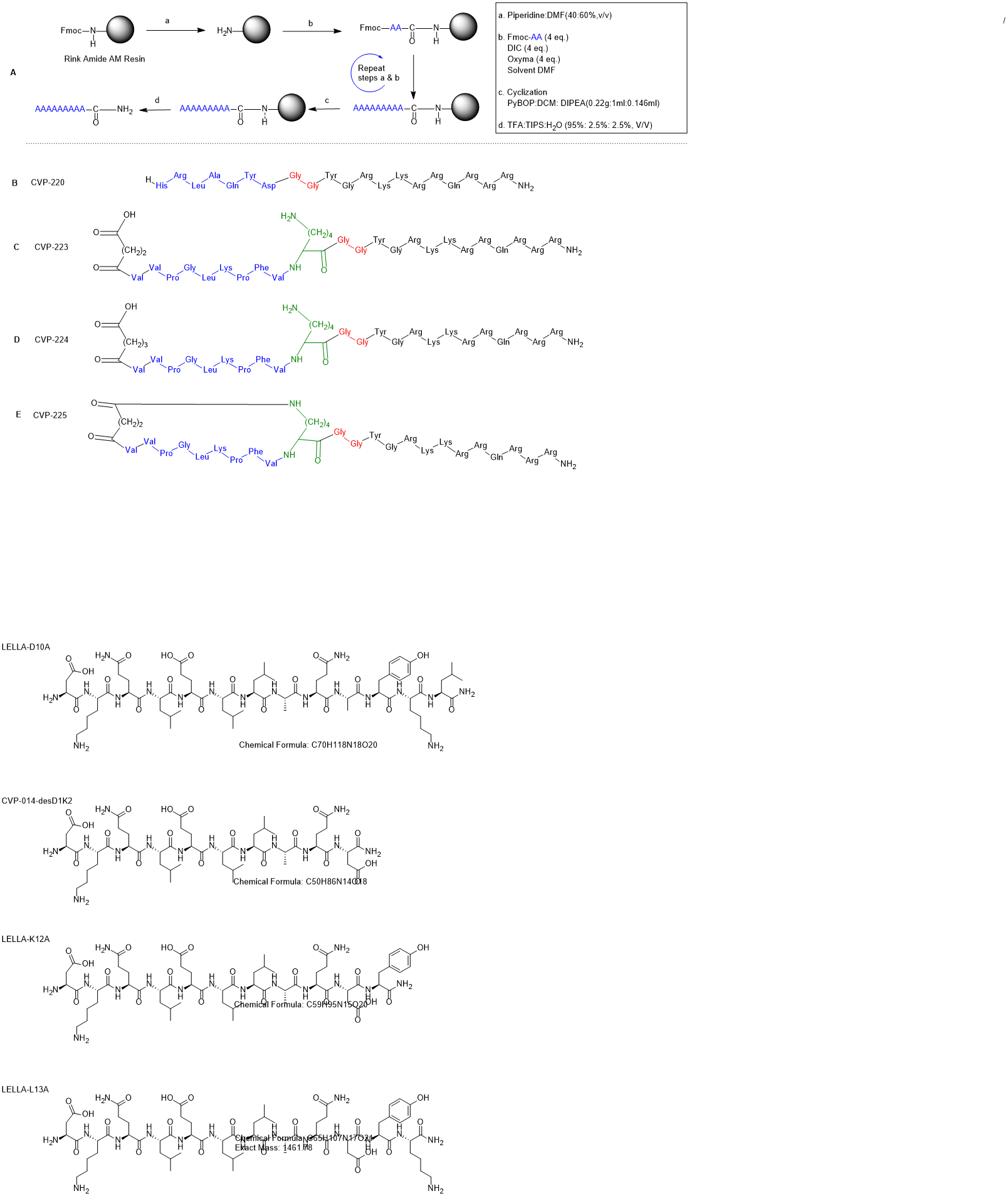

2.
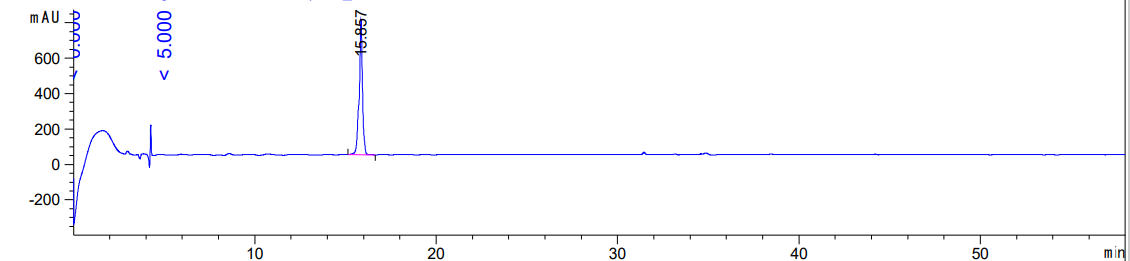


**C.**
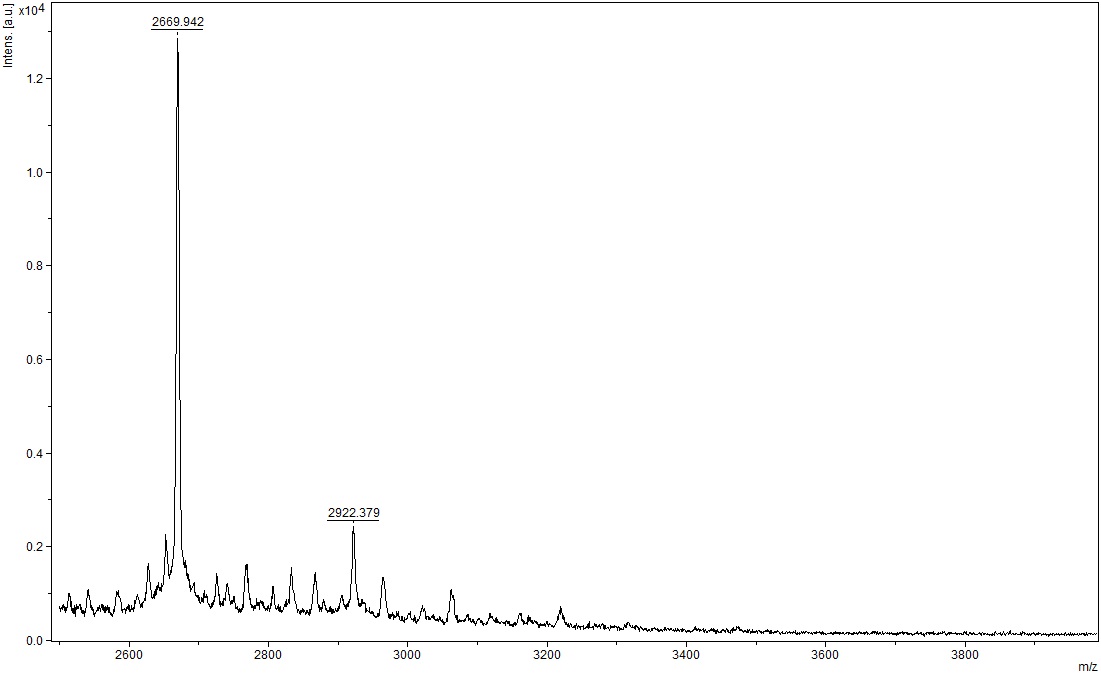


**Supplementary Figure 7**. Synthesis and characterization of CVP-242. (**A**) Structure of the peptide CVP-242. The bioactive peptide cargo (blue), and a short Gly-Gly spacer (red) conjugated with the TAT sequence (black) are highlighted with different colors. (**B**) HPLC Chromatography diagram of CVP-242. (**C**) Matrix-assisted laser desorption/ionization (MALDI) of CVP-242.

1.
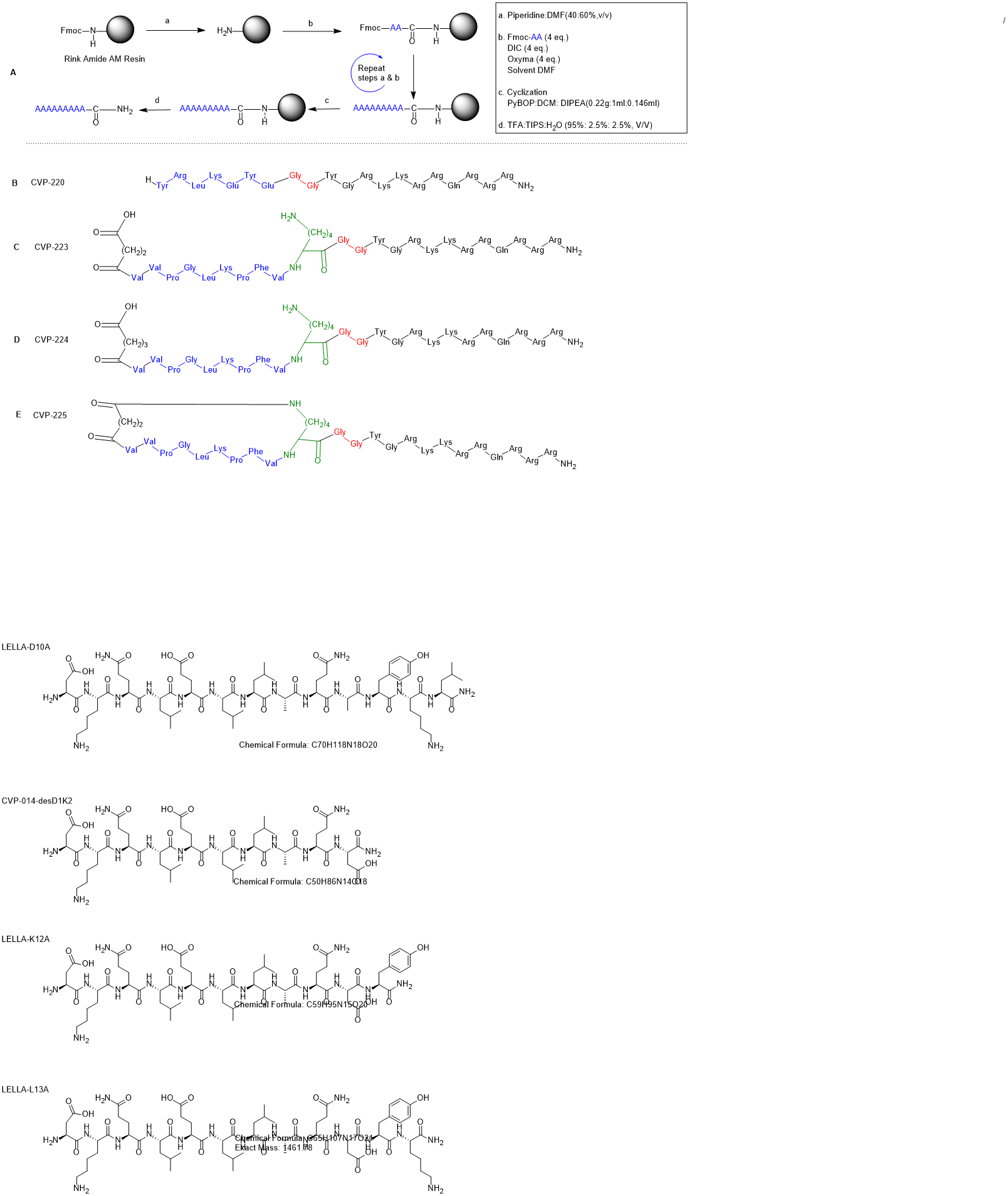

2.
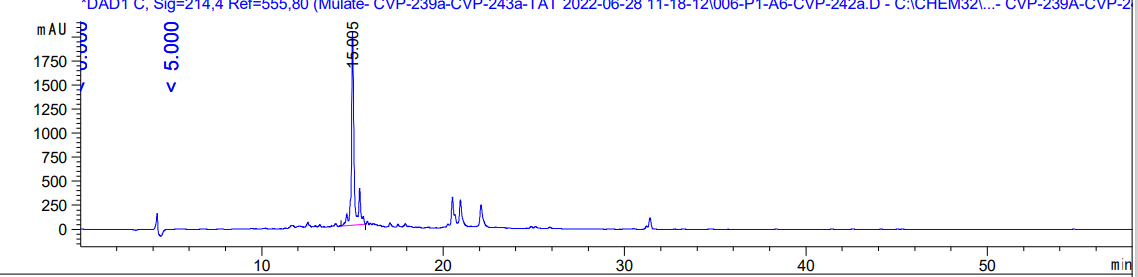

3.
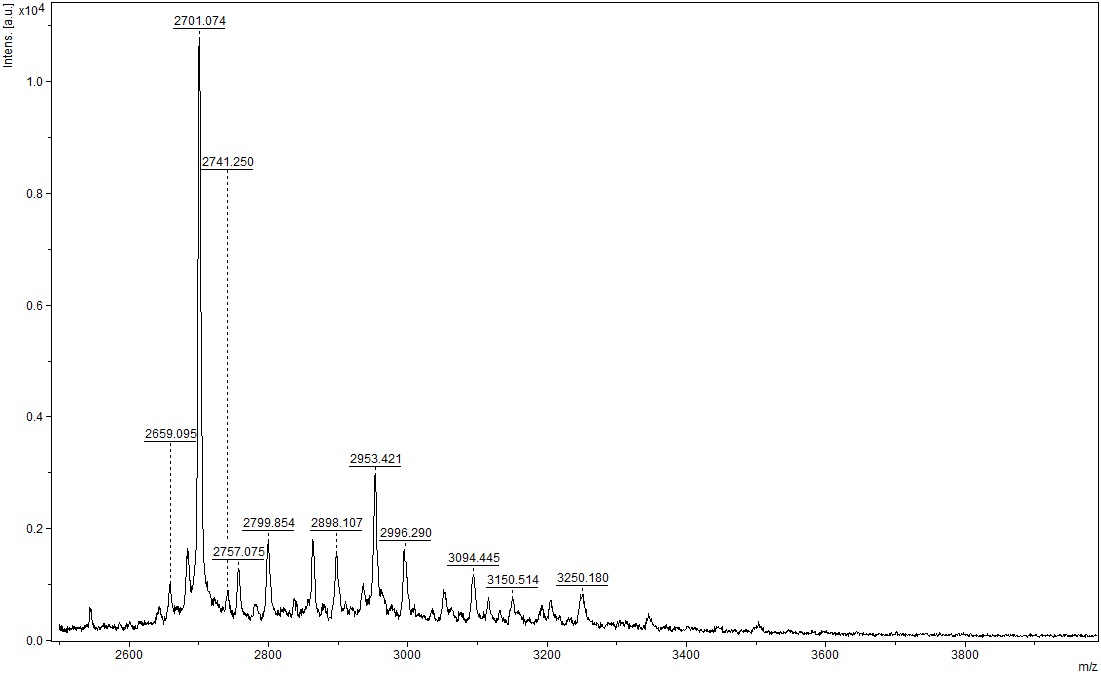


**Supplementary Table 3**. Characterizations of the developed peptides CVP-241, and CVP-242

| **Peptide Name** | **Cargo** | **Linker** | **Carrier (Cell-penetrating peptide)** | **Calculated molecular mass (Dalton)** | **Experimental molecular mass (Dalton)^a^** | **Purity HPLC (%)** |
| --- | --- | --- | --- | --- | --- | --- |
| **CVP-241** | HRLAQYD | GG | YGRKKRRQRRR | 2598.9671 | 2669.9420 | 100 |
| **CVP-242** | YRLKEYE | GG | YGRKKRRQRRR | 2697.1088 | 2701.0740 | 100 |

^a^ by Matrix-assisted laser desorption/ionization (MALDI, *Supporting material)*

**Supplementary Figure 8.** Competitive binding studies. Fis1 (500 nM) was immobilized on the chip and Mid51 was sampled at different concentrations: 2.5 nM, 5 nM, 20 nM, 40 nM, 80 nM, 100 nM, 200 nM, 300 nM, 500 nM, and 600 nM. The analyte was mixed with peptide (250 µM). Using field-effect biosensing (FEB) technology, real-time data was monitored for peptide-protein binding studies. The Y-axis corresponds to the I-Response in biosensor units (BU), and the X-axis corresponds to the different time points and concentrations of the analyte in the experiment. **(A)** Graphical representation of Fis1/Mid51 and CVP-241 peptide competitive binding. **(B)** Graphical representation of Fis1/Mid51 and CVP-242 peptide competitive binding**.**

**A.**
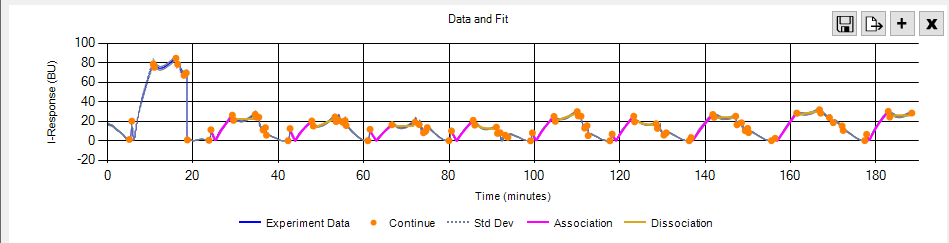


**B.**
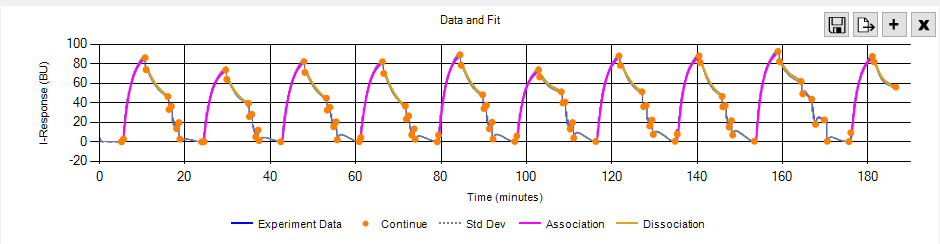


**Supplementary Figure 9.** Competitive inhibition studies to investigate peptide inhibition activities against Mid51/Fis1 interaction, *in vitro*. The Y-axis corresponds to the I-Response in biosensor units (BU), and the X-axis corresponds to the different concentrations of the analyte in the experiment (**A**) The inhibition activity of CVP-241 peptide; (**B**) The inhibition activity of CVP-242 peptide. Data are presented as mean ± SD (n = 3).

**A**. B**.**

**Supplementary Table 4**. Summarized results of peptide treatment effects. H9c2 cardiomyocytes were treated with Cobalt (II) and peptides (1 µM each). Cell viability was measured by XTT release. One-way variance analysis (ANOVA) with post-hoc Duncan analysis was done. Cell viability assay values are from independent experiments.

| Assay | Values | Treatments |  |  |
| --- | --- | --- | --- | --- |
|  |  | CoCl_2_ | CVP-241 | CVP-242 |
| XTT | Mean (n=8) | 60.00 | 70.61 | 69.98 |
|  | SD | 0.00 | 8.55 | 8.40 |
|  | SEM | 0.00 | 1.07 | 1.05 |
|  | P-value |  | 0.0029 | <0.0043 |
